# Genome-wide association studies in oesophageal adenocarcinoma and Barrett's oesophagus: a large-scale meta-analysis

**DOI:** 10.1016/S1470-2045(16)30240-6

**Published:** 2016-10

**Authors:** Puya Gharahkhani, Rebecca C Fitzgerald, Thomas L Vaughan, Claire Palles, Ines Gockel, Ian Tomlinson, Matthew F Buas, Andrea May, Christian Gerges, Mario Anders, Jessica Becker, Nicole Kreuser, Tania Noder, Marino Venerito, Lothar Veits, Thomas Schmidt, Hendrik Manner, Claudia Schmidt, Timo Hess, Anne C Böhmer, Jakob R Izbicki, Arnulf H Hölscher, Hauke Lang, Dietmar Lorenz, Brigitte Schumacher, Andreas Hackelsberger, Rupert Mayershofer, Oliver Pech, Yogesh Vashist, Katja Ott, Michael Vieth, Josef Weismüller, Markus M Nöthen, Barrett's and Esophageal Adenocarcinoma Consortium (BEACON), Esophageal Adenocarcinoma GenEtics Consortium (EAGLE), Wellcome Trust Case Control Consortium 2 (WTCCC2), Stephen Attwood, Hugh Barr, Laura Chegwidden, John de Caestecker, Rebecca Harrison, Sharon B Love, David MacDonald, Paul Moayyedi, Hans Prenen, R G Peter Watson, Prasad G Iyer, Lesley A Anderson, Leslie Bernstein, Wong-Ho Chow, Laura J Hardie, Jesper Lagergren, Geoffrey Liu, Harvey A Risch, Anna H Wu, Weimin Ye, Nigel C Bird, Nicholas J Shaheen, Marilie D Gammon, Douglas A Corley, Carlos Caldas, Susanne Moebus, Michael Knapp, Wilbert H M Peters, Horst Neuhaus, Thomas Rösch, Christian Ell, Stuart MacGregor, Paul Pharoah, David C Whiteman, Janusz Jankowski, Johannes Schumacher

**Affiliations:** a Statistical Genetics, QIMR Berghofer Medical Research Institute, Brisbane, QLD, Australia; b Cancer Control, QIMR Berghofer Medical Research Institute, Brisbane, QLD, Australia; c Medical Research Council (MRC) Cancer Unit, Hutchison-MRC Research Centre and University of Cambridge, Cambridge, UK; d Division of Public Health Sciences, Fred Hutchinson Cancer Research Center, Seattle, WA, USA; e Wellcome Trust Centre for Human Genetics, University of Oxford, Oxford, UK; f Centre for Statistics in Medicine, NDORMS, University of Oxford, Oxford, UK; g Department of Visceral, Transplant, Thoracic and Vascular Surgery, University Hospital of Leipzig, Leipzig, Germany; h Department of Medicine II, Sana Klinikum, Offenbach, Germany; i Department of Internal Medicine, Evangelisches Krankenhaus, Düsseldorf, Germany; j Department of Interdisciplinary Endoscopy, University Hospital Hamburg-Eppendorf, Hamburg, Germany; k Department of Gastroenterology and Interdisciplinary Endoscopy, Vivantes Wenckebach-Klinikum, Berlin, Germany; l Institute of Human Genetics, and Department of Genomics, Life & Brain Center, University of Bonn, Bonn, Germany; m Department of Gastroenterology, Hepatology and Infectious Diseases, Otto-von-Guericke University Hospital, Magdeburg, Germany; n Institute of Pathology, Klinikum Bayreuth, Bayreuth, Germany; o Department of General, Visceral and Transplantation Surgery, University of Heidelberg, Heidelberg, Germany; p Department of Internal Medicine II, Horst Schmidt Kliniken Hospital, Wiesbaden, Germany; q Department of General, Visceral and Cancer Surgery, University of Cologne, Cologne, Germany; r Department of General, Visceral and Thoracic Surgery, University Medical Center Hamburg-Eppendorf, University of Hamburg, Hamburg, Germany; s Department of General, Visceral and Transplant Surgery, University Medical Center, University of Mainz, Mainz, Germany; t Department of General and Visceral Surgery, Sana Klinikum, Offenbach, Germany; u Department of Internal Medicine and Gastroenterology, Elisabeth Hospital, Essen, Germany; v Gastropraxis, Wiesbaden, Germany; w Gastroenterologie am Burgweiher, Bonn, Germany; x Department of Gastroenterology and Interventional Endoscopy, St John of God Hospital, Regensburg, Germany; y Department of Visceral Surgery, Kantonsspital Aarau AG, Aarau, Switzerland; z Department of General, Visceral and Thorax Surgery, RoMed Klinikum Rosenheim, Rosenheim, Germany; aa Gastroenterologische Gemeinschaftspraxis, Koblenz, Germany; ab Centre For Integrated Health Care Research, Durham University, Durham, UK; ac Gloucestershire Royal Hospital, Gloucester, UK; ad Plymouth University Peninsula School of Medicine and Dentistry, Plymouth, UK; ae Digestive Diseases Centre, University Hospitals of Leicester, Leicester, UK; af Department of Cellular Pathology, Leicester Royal Infirmary, Leicester, UK; ag Department of Oral Biological and Medical Sciences, University of British Columbia, Vancouver, BC, Canada; ah Department of Medicine, McMaster University, Hamilton, ON, Canada; ai Department of Gastroenterology, University Hospitals Gasthuisberg, Leuven, Belgium; aj Queen's University Belfast, Centre of Medical Education, Royal Victoria Hospital, Belfast, UK; ak Division of Gastroenterology and Hepatology, Department of Internal Medicine, Mayo Clinic, Rochester, MN, USA; al Centre for Public Health, Queen's University Belfast, Belfast, UK; am Department of Population Sciences, Beckman Research Institute and City of Hope Comprehensive Cancer Center, Duarte, CA, USA; an Department of Epidemiology, MD Anderson Cancer Center, Houston, TX, USA; ao Division of Epidemiology, University of Leeds, Leeds, UK; ap Department of Molecular Medicine and Surgery, Karolinska Institute, Stockholm, Sweden; aq Department of Medical Epidemiology and Biostatistics, Karolinska Institute, Stockholm, Sweden; ar Division of Cancer Studies, King's College London, London, UK; as Pharmacogenomic Epidemiology, Ontario Cancer Institute, Toronto, ON, Canada; at Department of Chronic Disease Epidemiology, Yale School of Public Health, New Haven, CT, USA; au Department of Preventive Medicine, University of Southern California/Norris Comprehensive Cancer Center, Los Angeles, CA, USA; av Department of Oncology, Medical School, University of Sheffield, Sheffield, UK; aw Division of Gastroenterology and Hepatology, University of North Carolina School of Medicine, University of North Carolina, Chapel Hill, North Carolina, USA; ax Department of Epidemiology, University of North Carolina, Chapel Hill, North Carolina, USA; ay Division of Research, and San Francisco Medical Center, Kaiser Permanente Northern California, Oakland, CA, USA; az Department of Oncology, and Cancer Research UK Cambridge Institute, University of Cambridge, Cambridge, UK; ba Centre of Urban Epidemiology, Institute of Medical Informatics, Biometry and Epidemiology, University of Essen, Essen, Germany; bb Institute for Medical Biometry, Informatics, and Epidemiology, University of Bonn, Bonn, Germany; bc Department of Gastroenterology, Radboud University Nijmegen Medical Center, Nijmegen, Netherlands; bd Centre for Cancer Genetic Epidemiology, Department of Oncology, University of Cambridge, Cambridge, UK; be University of Central Lancashire, Westlakes Science and Technology Park, Moor Row, UK; bf Warwick Medical School, University of Warwick, Warwick, UK

## Abstract

**Background:**

Oesophageal adenocarcinoma represents one of the fastest rising cancers in high-income countries. Barrett's oesophagus is the premalignant precursor of oesophageal adenocarcinoma. However, only a few patients with Barrett's oesophagus develop adenocarcinoma, which complicates clinical management in the absence of valid predictors. Within an international consortium investigating the genetics of Barrett's oesophagus and oesophageal adenocarcinoma, we aimed to identify novel genetic risk variants for the development of Barrett's oesophagus and oesophageal adenocarcinoma.

**Methods:**

We did a meta-analysis of all genome-wide association studies of Barrett's oesophagus and oesophageal adenocarcinoma available in PubMed up to Feb 29, 2016; all patients were of European ancestry and disease was confirmed histopathologically. All participants were from four separate studies within Europe, North America, and Australia and were genotyped on high-density single nucleotide polymorphism (SNP) arrays. Meta-analysis was done with a fixed-effects inverse variance-weighting approach and with a standard genome-wide significance threshold (p<5 × 10^−8^). We also did an association analysis after reweighting of loci with an approach that investigates annotation enrichment among genome-wide significant loci. Furthermore, the entire dataset was analysed with bioinformatics approaches—including functional annotation databases and gene-based and pathway-based methods—to identify pathophysiologically relevant cellular mechanisms.

**Findings:**

Our sample comprised 6167 patients with Barrett's oesophagus and 4112 individuals with oesophageal adenocarcinoma, in addition to 17 159 representative controls from four genome-wide association studies in Europe, North America, and Australia. We identified eight new risk loci associated with either Barrett's oesophagus or oesophageal adenocarcinoma, within or near the genes *CFTR* (rs17451754; p=4·8 × 10^−10^), *MSRA* (rs17749155; p=5·2 × 10^−10^), *LINC00208* and *BLK* (rs10108511; p=2·1 × 10^−9^), *KHDRBS2* (rs62423175; p=3·0 × 10^−9^), *TPPP* and *CEP72* (rs9918259; p=3·2 × 10^−9^), *TMOD1* (rs7852462; p=1·5 × 10^−8^), *SATB2* (rs139606545; p=2·0 × 10^−8^), and *HTR3C* and *ABCC5* (rs9823696; p=1·6 × 10^−8^). The locus identified near *HTR3C* and *ABCC5* (rs9823696) was associated specifically with oesophageal adenocarcinoma (p=1·6 × 10^−8^) and was independent of Barrett's oesophagus development (p=0·45). A ninth novel risk locus was identified within the gene *LPA* (rs12207195; posterior probability 0·925) after reweighting with significantly enriched annotations. The strongest disease pathways identified (p<10^−6^) belonged to muscle cell differentiation and to mesenchyme development and differentiation.

**Interpretation:**

Our meta-analysis of genome-wide association studies doubled the number of known risk loci for Barrett's oesophagus and oesophageal adenocarcinoma and revealed new insights into causes of these diseases. Furthermore, the specific association between oesophageal adenocarcinoma and the locus near *HTR3C* and *ABCC5* might constitute a novel genetic marker for prediction of the transition from Barrett's oesophagus to oesophageal adenocarcinoma. Fine-mapping and functional studies of new risk loci could lead to identification of key molecules in the development of Barrett's oesophagus and oesophageal adenocarcinoma, which might encourage development of advanced prevention and intervention strategies.

**Funding:**

US National Cancer Institute, US National Institutes of Health, National Health and Medical Research Council of Australia, Swedish Cancer Society, Medical Research Council UK, Cambridge NIHR Biomedical Research Centre, Cambridge Experimental Cancer Medicine Centre, Else Kröner Fresenius Stiftung, Wellcome Trust, Cancer Research UK, AstraZeneca UK, University Hospitals of Leicester, University of Oxford, Australian Research Council.

## Introduction

Oesophageal adenocarcinoma is a fatal cancer that ranks eleventh in mortality among all malignant disorders.^1^ Although new treatment strategies—eg, neoadjuvant chemoradiotherapy—have improved survival, patients with oesophageal adenocarcinoma still have a poor prognosis.^2^ Barrett's oesophagus is the premalignant precursor of oesophageal adenocarcinoma and is characterised by a metaplastic change of the stratified squamous epithelium in the distal oesophagus to a glandular so-called intestinalised epithelium.^3^ The main risk factor for Barrett's oesophagus is gastro-oesophageal reflux, whereby gastric acid chronically damages the epithelium of the distal oesophagus.^3^ However, although Barrett's oesophagus has an estimated prevalence of up to 5·6% in the population,^4^ only a few patients with this disorder—roughly 0·12% every year—develop oesophageal adenocarcinoma.^5^ This low progression rate complicates clinical management of Barrett's oesophagus because no valid predictors for the transition from Barrett's oesophagus to oesophageal adenocarcinoma exist, and thus there are no effective surveillance and intervention strategies.

Barrett's oesophagus and oesophageal adenocarcinoma have heritable components with substantial overlap in the set of genes contributing to risk of each condition.^6^ However, genetic risk factors contributing specifically to Barrett's oesophagus or oesophageal adenocarcinoma alone might also exist. So far, genome-wide association studies have identified four loci within or near MHC, *FOXF1, GDF7*, and *TBX5* associated with the development of Barrett's oesophagus,^7^, ^8^ and four additional loci within or near *CRTC1, BARX1, FOXP1*, and *ALDH1A2* associated with development of both Barrett's oesophagus and oesophageal adenocarcinoma.^8^, ^9^ However, because of small sample sizes analysed so far, these loci account for only a part of the genetic variance of Barrett's oesophagus and oesophageal adenocarcinoma.^6^ Furthermore, these loci are insufficient to predict the transition from Barrett's oesophagus to oesophageal adenocarcinoma, because no specific marker for oesophageal adenocarcinoma has been identified up to now.

Therefore, our international consortium aimed to do a meta-analysis of all available datasets from genome-wide association studies for Barrett's oesophagus and oesophageal adenocarcinoma to identify additional genetic variants associated with risk for both disorders. Furthermore, we aimed to identify genetic variants that contribute specifically to risk for oesophageal adenocarcinoma and, thereby, might serve as markers for individualised surveillance and intervention strategies for Barrett's oesophagus. To our knowledge, our study is the first in which datasets from genome-wide association studies have been analysed using bioinformatics approaches to gain further information about the underlying genes and cellular pathways associated with Barrett's oesophagus and oesophageal adenocarcinoma.

**Research in context**

**Evidence before this study**

We searched PubMed on Feb 29, 2016, to identify genetic risk markers for Barrett's oesophagus and oesophageal adenocarcinoma identified through genome-wide association studies. We did not apply any publication date restrictions. The search was restricted to papers published in the English language. Search terms were: (“esophageal” OR “oesophageal” OR “esophagus” OR “oesophagus”) AND (“Barrett's” OR “adenocarcinoma”) AND (“genome wide association study” OR “GWAS”). Three genome-wide association studies have been published to date and have led to the identification of eight genetic risk loci contributing to both Barrett's oesophagus and oesophageal adenocarcinoma. These encouraging findings, however, account for only a part of the genetic risk for Barrett's oesophagus and oesophageal adenocarcinoma. In particular, no variants have been identified so far that contribute solely to development of oesophageal adenocarcinoma and, thereby, might serve as markers for more effective surveillance and intervention strategies for Barrett's oesophagus.

**Added value of this study**

Within an international consortium, we did a meta-analysis of four datasets available to date from genome-wide association studies, totalling more than 27 000 individuals. We identified nine new risk loci for Barrett's oesophagus or oesophageal adenocarcinoma, or both, which represents a doubling of the number of known risk loci. The most strongly associated new risk variant is located within *CFTR*, mutations of which lead to cystic fibrosis. Patients with cystic fibrosis show highly increased incidence of gastro-oesophageal reflux, and this reflux represents the main risk factor for Barrett's oesophagus and oesophageal adenocarcinoma. Therefore, our data suggest that cystic fibrosis, Barrett's oesophagus, and oesophageal adenocarcinoma might have a common pathophysiological feature of gastro-oesophageal reflux, with *CFTR* playing an important part in this process. We also identified a risk variant near *HTR3C/ABCC5* that was associated solely with development of oesophageal adenocarcinoma. This variant might constitute a novel marker for the prediction of transition from Barrett's oesophagus to oesophageal adenocarcinoma.

**Implications of all the available evidence**

Identification of novel risk loci and cellular pathways provides further insights into the causes of Barrett's oesophagus and oesophageal adenocarcinoma and impetus for further functional studies. The marker specific to oesophageal adenocarcinoma should help to identify patients at higher risk for the transition from Barrett's oesophagus to oesophageal adenocarcinoma. Together, this information should lead to better molecular treatments and individualised prevention and intervention strategies for clinical management of Barrett's oesophagus and oesophageal adenocarcinoma.

## Methods

### Study design and participants

We obtained genome-wide genotype data for patients with Barrett's oesophagus, individuals with oesophageal adenocarcinoma, and representative controls from four genome-wide association studies in Europe, North America, and Australia:^7^, ^8^, ^9^ the Barrett's and Esophageal Adenocarcinoma Consortium (BEACON) study; and studies from Bonn, Cambridge, and Oxford (appendix pp 5–6, 11). Data from the Bonn study are unpublished; the Oxford study did not contribute data for patients with oesophageal adenocarcinoma. All participants were of European ancestry, and DNA samples extracted from blood or saliva were genotyped on high-density single nucleotide polymorphism (SNP) arrays (Illumina, San Diego, CA, USA).

Patients with Barrett's oesophagus were identified by histopathological diagnosis of intestinal metaplasia, and individuals with oesophageal adenocarcinoma had a histopathological diagnosis of adenocarcinoma. We excluded all other patients. Informed consent was obtained in the four studies from all participants and ethics approval was obtained from the ethics boards of every participating institution.

### Procedures

We did a quality control assessment of genotyped markers, genotyped individuals, and the imputation, using the same protocol at all participating sites. We used PLINK version 1.90^10^ for quality control. We removed all individuals with more than 3% of missing genotypes; SNPs with a successful genotyping rate of less than 97%; SNPs with a minor allele frequency less than 0·01; SNPs with a p value of less than 0·0001 in controls and less than 5 × 10^−10^ in patients for Hardy-Weinberg equilibrium; and SNPs with a significant (p<0·001) difference in missingness between cases and controls. Based on identity by descent calculated from autosomal markers, we removed one of each pair of individuals with high levels of relatedness (p-hat>0·2) and a higher proportion of missing genotypes. We also removed participants who lay beyond six SDs from the mean of the first two genotypic principal components of the 1000 Genomes European descent population.^11^

For the imputation, we used SHAPEIT version 2.12^12^ for phasing of the genotyped SNPs and IMPUTE2 version 2.3.1^13^, ^14^ for imputation of missing SNPs, using the 1000 Genomes Phase 1 haplotypes (June, 2014 release) as a reference panel.^15^ We did the imputation in 5 Mb sections. We set a 250 kb buffer flanking the imputation sections and an effective size of the sampled population of 20 000, as recommended for IMPUTE2 version 2.3.1.^13^, ^14^

### Statistical analysis

We did association testing for Barrett's oesophagus and oesophageal adenocarcinoma as separate disorders. We then repeated the analysis after combining the two groups of patients into a single group. We assessed associations in SNPTEST version 2.5.2,^16^ adjusted for sex and study-specific top principal components, under an additive genetic model using dosage scores (based on the probabilities for each of the three possible genotypes of every SNP) obtained from the imputation. Dosage scores account for imputation uncertainty in the association analysis, by contrast with the best-guess approach, whereby the most probable genotype of every SNP obtained from imputation is regarded as the actual genotype for that SNP. We calculated the genomic inflation factor lambda (λ) to ensure that the results were not affected by model mis-specification. A high inflation factor might indicate presence of population stratification, unknown familial relationships, undetected sample duplications, technical problems with the data, or application of incorrect statistical methods.

We analysed SNPs that passed the post-imputation quality control assessment in every study (imputation quality score >0·4, minor allele frequency >0·001) and were present in at least three studies of Barrett's oesophagus and two studies of oesophageal adenocarcinoma. An imputation quality score greater than 0·4 ensures that SNPs that were not well imputed were excluded, and a minor allele frequency greater than 0·001 ensures that SNPs that were not common in our study population were excluded from the analysis (appendix pp 5–6). We did the meta-analysis with the fixed-effects inverse variance-weighting approach in METAL version 2011-03-25,^17^ with a standard genome-wide significant threshold of 5 × 10^−8^.

We investigated the presence of genetic heterogeneity between studies with the *I*^2^ statistic, and we calculated p values for heterogeneity with Cochran's Q test, as implemented in METAL version 2011-03-25.^17^ Presence of genetic heterogeneity indicates that effect sizes are not similar between studies, emphasising the possibility of a distribution of true effect sizes between studies. Random-effects meta-analysis deals with this situation by decomposing the observed variance into its two components, within and between study variance, and uses both components for weighting. We did random-effects meta-analysis in PLINK version 1.90^10^ for all genome-wide significant SNPs that showed significant genetic heterogeneity (p for heterogeneity <0·05).

We created Q-Q and Manhattan plots for the meta-analysis in R. We used LocusZoom version 1.1^18^ to create regional association plots for genome-wide significant results.

To investigate whether independent associations exist in regions of genome-wide significance, we did association analyses conditioned on the strongest associated SNP in every region (1 Mb either side of the top SNPs) with meta-analysis summary statistics and the approach implemented in GCTA version 1.25.2.^19^ This approach uses both summary-level statistics from genome-wide association studies and estimated linkage disequilibrium from a reference sample (the imputed BEACON data in this study) to investigate whether single or multiple independent associations exist for every locus.

Because some SNPs could be associated with Barrett's oesophagus and oesophageal adenocarcinoma but not meet the genome-wide significance threshold because of insufficient statistical power (ie, SNPs with small effect sizes cannot be detected in our current sample size using stringent criteria for significance), we used a new approach^20^ in which functional annotation information from genome-wide significant loci is used to reweight the results. Incorporating functional annotation information to reweight data from genome-wide association studies could result in identification of new risk loci that otherwise might not reach the genome-wide significance threshold in standard genome-wide association studies. This approach, which is implemented in fgwas version 1.0,^20^ is capable of identifying additional high-confidence risk loci, resulting in a roughly 5% increase in the number of identified loci when tested on previously published data from genome-wide association studies.^20^ We looked at enrichment of 450 genomic annotations as implemented in fgwas version 1.0^20^ (default settings: 5000 SNPs per window). We derived the best annotations from genome-wide significant loci in the Barrett's oesophagus and oesophageal adenocarcinoma combined analysis. We first considered annotations separately to see if they were individually significant. Some annotations were correlated and, hence, we built a model by adding terms sequentially in decreasing order of significance until no more annotations significantly (p<0·05) improved the log-likelihood of the model. We then applied the cross-validation approach implemented in fgwas version 1.0 to ensure no over-fitting in the final model. We used this final Bayesian model to derive a prior distribution for the remainder of the genome. We calculated the posterior probability of association based on the derived prior distribution. A posterior probability greater than 0·9 in this approach performed similarly to the genome-wide significance threshold in genome-wide association studies (p<5 × 10^−8^) based on the analysis^20^ of previously published genome-wide association studies.^20^

We did gene-based association tests with the approach implemented in VEGAS version 2,^21^ a simulation-based approach that combines the test statistics for single variants within gene boundaries while accounting for linkage disequilibrium between markers. We set the Bonferroni-corrected threshold for gene-wide significance to a p value of less than 2·8 × 10^−6^ (considering 17 787 autosomal genes used in VEGAS version 2).

We analysed pathways and tissue enrichment with methods implemented in DEPICT version 1.1.^22^ The preference is to use genome-wide significant SNPs as long as at least ten independent loci are available. However, because of the polygenic basis of complex traits, restricting the pathways analysis to only genome-wide significant SNPs might result in some informative data being missed. This omission is because many SNPs that do not meet the genome-wide significance threshold might still be associated with either Barrett's oesophagus or oesophageal adenocarcinoma (or both), but might not be detected because of insufficient statistical power. Accordingly, we included loci from the combined Barrett's oesophagus and oesophageal adenocarcinoma meta-analysis that achieved one of three p value thresholds (p<5 × 10^−8^, p<10^−6^, and p<10^−4^) for pathways analysis. We set the Bonferroni-corrected threshold for pathways analysis at a p value of less than 1·15 × 10^−6^ (considering multiple testing with the three p-value thresholds and assuming all 14 463 pathways used in DEPICT version 1.1 are independent) and a false discovery rate of less than 0·05. Similarly, we set the Bonferroni-corrected threshold for tissue-enrichment analysis to a p value of less than 8 × 10^−6^ (considering multiple testing with the three p-value thresholds and assuming that gene expression in all 209 tissue and cell samples used in DEPICT version 1.1 is independent) and a false discovery rate less than 0·05.

We did bioinformatics analyses as described in the appendix (p 6). We investigated whether published risk loci for gastro-oesophageal reflux-predisposing traits (eg, body-mass index [BMI] and obesity), which have shown genome-wide significant associations,^23^ represent risk loci for Barrett's oesophagus and oesophageal adenocarcinoma. We also estimated the peak SNPs identified in this study in the genome-wide association analysis for BMI undertaken by the Genetic Investigation of ANthropometric Traits (GIANT) consortium.^24^ Additional details of methods used for functional annotation enrichment analysis, gene-based analysis, and tissue enrichment analysis are in the appendix (p 7).

### Role of the funding source

The funders had no role in study design, data collection, data analysis, data interpretation, or writing of the report. The corresponding author had full access to all data in the study, except personal identifying information, and had final responsibility for the decision to submit for publication.

## Results

6167 people with Barrett's oesophagus, 4112 individuals with oesophageal adenocarcinoma, and 17 159 representative controls from four genome-wide association studies in Europe, North America, and Australia were included in the meta-analysis. In total, 11 942 825 SNPs for Barrett's oesophagus, 13 074 274 for oesophageal adenocarcinoma, and 11 951 684 for both Barrett's oesophagus and oesophageal adenocarcinoma were used for the meta-analysis of genome-wide association studies. Q-Q and Manhattan plots from the separate Barrett's oesophagus and oesophageal adenocarcinoma meta-analyses, and from the combined Barrett's oesophagus and oesophageal adenocarcinoma meta-analysis, are shown in the appendix (pp 8–9). The scaled genomic inflation factor lambda (λ) was 1·043 for the Barrett's oesophagus meta-analysis, 1·005 for the oesophageal adenocarcinoma meta-analysis, and 1·049 for the combined Barrett's oesophagus and oesophageal adenocarcinoma meta-analysis.

Five genome-wide significant associated loci (p<5 × 10^−8^) were identified for Barrett's oesophagus alone, of which three were not previously reported (appendix p 11). Moreover, five genome-wide significant associated loci (p<5 × 10^−8^) for oesophageal adenocarcinoma alone were identified, of which four were previously unreported (appendix p 12). The combined meta-analysis for Barrett's oesophagus and oesophageal adenocarcinoma identified 14 genome-wide significant associated loci (p<5 × 10^−8^), of which seven were previously unreported (table). Of note, all seven new genome-wide significant loci from the separate Barrett's oesophagus and oesophageal adenocarcinoma meta-analyses were also identified in the combined meta-analysis except for one locus on chromosome 3q27 near *HTR3C* and *ABCC5* (rs9823696) that was only recorded in the oesophageal adenocarcinoma meta-analysis and, therefore, was specific for this disorder (risk for oesophageal adenocarcinoma: odds ratio [OR] 1·17, 95% CI 1·11–1·24; p=1·64 × 10^−8^; risk for Barrett's oesophagus: 1·02, 0·97–1·06; p=0·45). By contrast, all risk loci identified for Barrett's oesophagus were also associated with oesophageal adenocarcinoma (at least p<0·02; appendix p 13).

**Figure 1:**
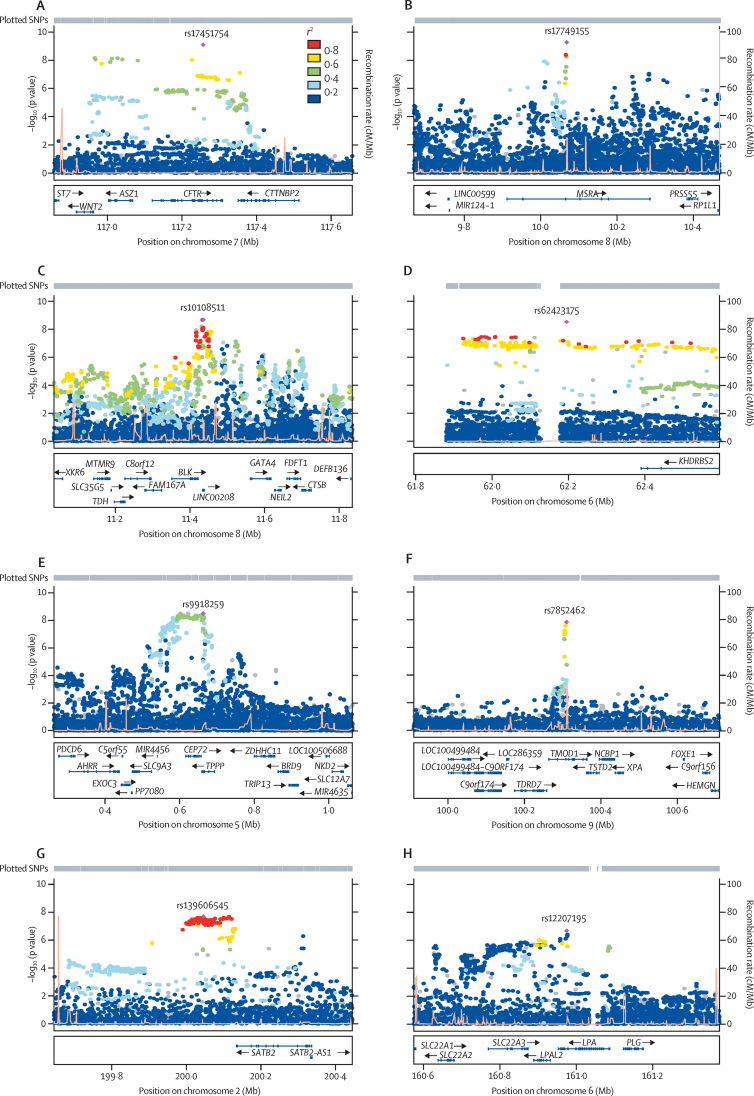
Regional plots for loci meeting the threshold for genome-wide significance in both Barrett's oesophagus and oesophageal adenocarcinoma Regional associations for the most significantly associated single nucleotide polymorphisms (SNPs; marked as solid purple diamonds) in the combined Barrett's oesophagus and oesophageal adenocarcinoma meta-analysis (includes 10 279 patients with Barrett's oesophagus and oesophageal adenocarcinoma and 17 159 controls). Pairwise correlations (*r*^2^) between the top SNP and the other SNPs in a 400 kb flanking region are illustrated by different colours. Grey dots denote the SNPs that were not present in the reference panel that was used to calculate linkage disequilibrium between SNPs. Light orange spikes show estimated recombination rates. (A) rs17451754 on chromosome 7q31 within *CFTR*. (B) rs17749155 on chromosome 8p23 within *MSRA*. (C) rs10108511 on chromosome 8p23 within *LINC00208* and *BLK*. (D) rs62423175 on chromosome 6q11 near *KHDRBS2* and *MTRNR2L9*. (E) rs9918259 on chromosome 5p15 within *TPPP* and *CEP72*. (F) rs7852462 on chromosome 9q22 within *TMOD1*. (G) rs139606545 on chromosome 2q33 near *SATB2*. (H) rs12207195 on chromosome 6q26 within *LPA*. cM=centimorgan.

Regional association results for all novel Barrett's oesophagus and oesophageal adenocarcinoma loci are shown in figure 1. The most strongly associated SNPs were rs17451754 on chromosome 7q31 within *CFTR* (p=4·77 × 10^−10^; figure 1A), rs17749155 on chromosome 8p23 within *MSRA* (p=5·21 × 10^−10^; figure 1B), rs10108511 on chromosome 8p23 within *LINC00208* and *BLK* (p=2·12 × 10^−9^; figure 1C), rs62423175 on chromosome 6q11 near *KHDRBS2* and *MTRNR2L9* (p=2·95 × 10^−9^; figure 1D), rs9918259 on chromosome 5p15 within *TPPP* and *CEP72* (p=3·23 × 10^−9^; figure 1E), rs7852462 on chromosome 9q22 within *TMOD1* (p=1·49 × 10^−8^; figure 1F), and rs139606545 on chromosome 2q33 near *SATB2* (p=2·02 × 10^−8^; figure 1G). We identified an additional risk locus for Barrett's oesophagus and oesophageal adenocarcinoma (rs12207195) at the gene *LPA* on chromosome 6q26 (figure 1H). Although rs12207195 did not reach genome-wide significance in the frequentist analysis (p=2·1 × 10^−7^), the posterior probability for the region containing *LPA* was 0·925 in the empirical Bayesian approach (compared with 0·863 without weighting by annotation; appendix p 7), corresponding to p<5 × 10^−8^ in the frequentist inference. The appendix (p 13) shows the association results of the top associated SNPs from the combined meta-analysis in the separate Barrett's oesophagus and oesophageal adenocarcinoma analyses.

**Figure 2:**
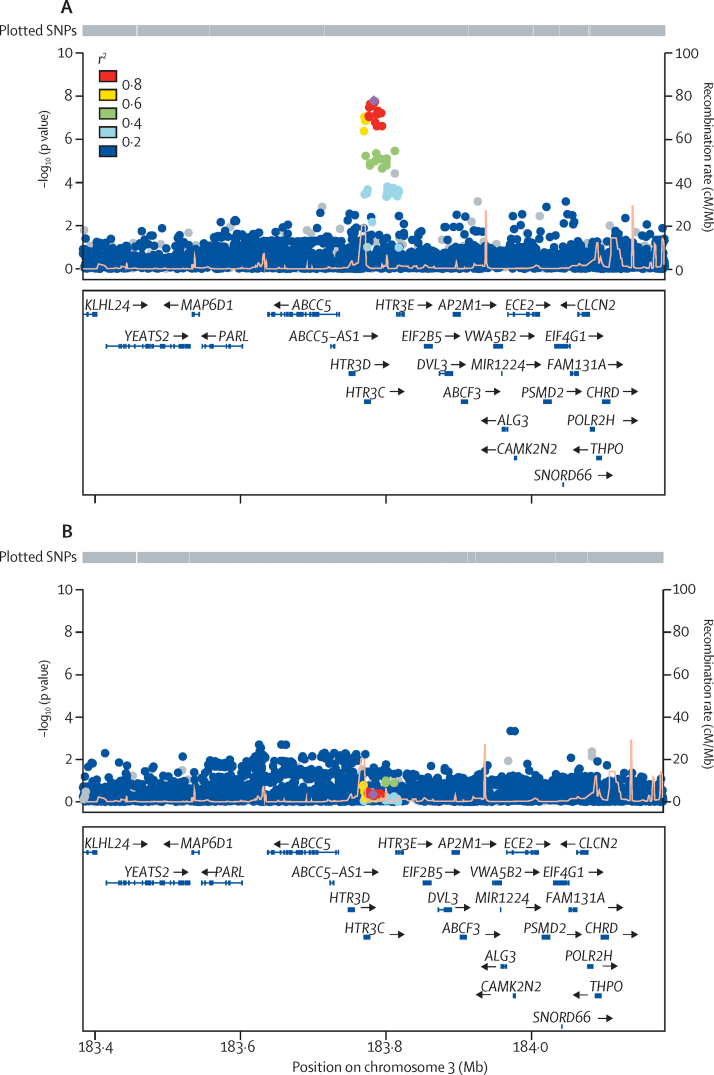
Regional plots for the oesophageal adenocarcinoma-specific locus rs9823696 near *HTR3C* and *ABCC5* Regional associations for the most significantly associated single nucleotide polymorphism (SNP; marked as a solid purple diamond), rs9823696, in the oesophageal adenocarcinoma meta-analysis. Pairwise correlations (*r*^2^) between the top SNP and the other SNPs in a 400 kb flanking region are illustrated by different colours. Grey dots denote the SNPs that were not present in the reference panel that was used to calculate linkage disequilibrium between SNPs. Light orange spikes show estimated recombination rates. (A) Genome-wide significance in 4112 patients with oesophageal adenocarcinoma and 13 663 controls (p=1·64 × 10^−8^). (B) Not significant in 6167 patients with Barrett's oesophagus and 17 159 controls (p=0·45). cM=centimorgan.

Figure 2 shows regional association results for the oesophageal adenocarcinoma-specific locus near *HTR3C* and *ABCC5* (rs9823696) in oesophageal adenocarcinoma and in Barrett's oesophagus. Although we did not identify any secondary peaks (ie, associations of SNPs with oesophageal adenocarcinoma and Barrett's oesophagus that were independent of the top hits) at genome-wide significance in the conditional association analysis of the combined meta-analysis, two loci (rs34817486 near *FOXF1-AS1* [also known as *FENDRR*] and *FOXF1,* and rs62331139 near *LPCAT1* and *SLC6A3*) showed some evidence of secondary peaks (p<10^−5^; appendix p 10).

Of the nine newly identified risk loci, only SNPs within or near *TPPP* and *CEP72* showed significant (p<0·05) heterogeneity for the magnitudes of association of SNPs between studies in the fixed-effects meta-analysis (heterogeneity *I*^2^=64·5 and p=0·0375 for rs9918259, the most significantly associated SNP at this locus; table). All studies in this meta-analysis showed the same direction of effect for risk alleles at this locus. However, the magnitude of association was larger in the Bonn study compared with the other studies—ie, the Bonn study OR was 1·43 (95% CI 1·25–1·64) for the risk allele of rs9918259, whereas it was 1·18 (1·08–1·29) in the BEACON study, 1·11 (0·98–2·49) in the Cambridge study, and 1·12 (0·99–1·28) in the Oxford study. Under a random-effects model, the SNP rs9918259 was less significantly associated with Barrett's oesophagus and oesophageal adenocarcinoma in the combined meta-analysis (p=4·7 × 10^−4^) than with the fixed effects meta-analysis (p=3·2 × 10^−9^). Consistent with p values for heterogeneity for the other risk loci (table), the magnitude and direction of effect were consistent between all studies for the remaining risk loci. Thus, we did not do a random-effects meta-analysis for these loci.

All previously reported genome-wide significant loci^7^, ^8^, ^9^—including *GDF7, ALDH1A2, TBX5, CRTC1, FOXP1, FOXF1,* and the MHC region (table)—were also associated with both Barrett's oesophagus and oesophageal adenocarcinoma at the genome-wide significance threshold. Only the *BARX1* locus^9^ did not meet the genome-wide significance threshold, but it still showed strong association with Barrett's oesophagus and oesophageal adenocarcinoma in the combined meta-analysis (p=6·2 × 10^−7^ for rs11789015). Apart from the risk loci identified in the single variant analysis, we did not identify other loci reaching gene-based genome-wide significance (p<2·8 × 10^−6^) after correction for genomic inflation in the gene-based association analysis (appendix p 7).

In the pathway analyses, no pathways were significantly associated with Barrett's oesophagus and oesophageal adenocarcinoma at the thresholds p<1·15 × 10^−6^ and false discovery rate <0·05 using SNPs satisfying p<5 × 10^−8^ and p<10^−6^ in the combined meta-analysis. However, for SNPs satisfying p<1 × 10^−4^ in the combined meta-analysis, four pathways were significantly associated with Barrett's oesophagus and oesophageal adenocarcinoma (appendix p 14): negative regulation of muscle-cell differentiation (GO:0051148); mesenchyme development (GO:0060485); BMPR2 PPI subnetwork (ENSG00000204217); and mesenchymal cell differentiation (GO:0048762). Separate Barrett's oesophagus and oesophageal adenocarcinoma pathways analyses with these thresholds did not identify any significant pathway. In tissue enrichment analyses, genes within the combined Barrett's oesophagus and oesophageal adenocarcinoma associated regions were highly expressed in the digestive system, as well as in the endocrine system, cardiovascular system, and in smooth muscle (appendix pp 7, 15).

None of the published genome-wide significant risk loci for BMI and obesity were associated with Barrett's oesophagus and oesophageal adenocarcinoma in the combined meta-analysis at the genome-wide significance level (data not shown). However, rs2898290 (within *LINC00208* and *BLK*), which is strongly associated with Barrett's oesophagus and oesophageal adenocarcinoma (p=1·2 × 10^−8^), showed some evidence of association with BMI in the GIANT study^24^ (p=0·001058).

The nine newly identified Barrett's oesophagus and oesophageal adenocarcinoma risk loci were characterised by analysis of multiple functional annotation databases (appendix pp 16–18). Many loci harbour genes expressed in the gastrointestinal tract and that have a role in oncogenesis. Furthermore, some of the identified risk variants—or variants that are highly correlated with them (*r*^2^>0·80)—represent expression quantitative trait loci that regulate the expression of genes within the regions. Moreover, several of the implicated risk variants change sequence motifs for protein binding sites and are located within DNAase hypersensitivity regions and within regions with enhancer or promoter motifs.

## Discussion

Our meta-analysis identified 16 independent risk loci for development of Barrett's oesophagus, oesophageal adenocarcinoma, or both, at the level of genome-wide significance. Nine loci had not been identified before; all previously reported risk loci were associated with both Barrett's oesophagus and oesophageal adenocarcinoma in our meta-analysis. Thus, our study has more than doubled the number of known risk loci for Barrett's oesophagus and oesophageal adenocarcinoma, which further exemplifies the scientific value of meta-analysis of genome-wide association studies through international collaborations. Moreover, we identified an oesophageal adenocarcinoma-specific risk locus that was independent of development of Barrett's oesophagus. The sample size of our meta-analysis was large enough to do a pathway analysis to investigate genetic pathways associated with development of Barrett's oesophagus and oesophageal adenocarcinoma. Our findings indicated that cellular pathways involved in muscle-cell differentiation and mesenchyme development and differentiation were implicated in causing Barrett's oesophagus and oesophageal adenocarcinoma.

Findings of the functional annotation database analysis of the newly identified Barrett's oesophagus and oesophageal adenocarcinoma risk loci exemplify how data from genome-wide association studies can uncover new causal and clinical aspects of Barrett's oesophagus and oesophageal adenocarcinoma (appendix pp 18–19). The newly identified risk locus with the strongest association with Barrett's oesophagus and oesophageal adenocarcinoma (p=4·8 × 10^−10^) was rs17451754 on chromosome 7q31. This SNP is located within intron 21 of the *CFTR* gene and affects a region marked by enhancer histone modifications in the gastrointestinal tract mucosa and by DNAse hypersensitivity.^25^ *CFTR* encodes an ATP-binding cassette membrane protein that functions as a chloride channel and is mutated in cystic fibrosis,^26^ the most common autosomal recessive disorder among people of European ancestry. Mutations in *CFTR* lead to secretions that are abnormally viscous and altered in their chemical composition, leading to severe dysfunction of the respiratory system and gastrointestinal tract. Up to 81% of patients with cystic fibrosis have gastro-oesophageal reflux, a major risk factor for Barrett's oesophagus and oesophageal adenocarcinoma, and more than 50% of these individuals are treated with proton-pump inhibitors in high-income countries.^27^ According to findings of a 20-year nationwide survey from the USA,^28^ incidence of cancer at the gastro-oesophageal junction is also increased among patients with cystic fibrosis, with evidence of Barrett's oesophagus in these patients. Although the cause of gastro-oesophageal reflux seems to differ between most patients with and without cystic fibrosis, the exact mechanism of reflux in patients with cystic fibrosis is still not understood fully. Favoured pathophysiological ideas about gastro-oesophageal reflux in patients with cystic fibrosis include lower inspiratory intrathoracic pressure with altered gastro-oesophageal pressure gradients,^29^ delayed gastric emptying,^30^ and impaired neutralisation of reflux-acidified oesophageal mucosa because of reduced bicarbonate secretion or hyperacidity of refluxed gastric contents.^31^ However, in view of the phenotypic overlap for gastro-oesophageal reflux and cystic fibrosis, and for gastro-oesophageal reflux and both Barrett's oesophagus and oesophageal adenocarcinoma, combined with the identification of *CFTR* risk variants in patients with Barrett's oesophagus and oesophageal adenocarcinoma, it seems plausible that a common pathophysiological mechanism for gastro-oesophageal reflux is triggered by *CFTR*. This idea underlines the importance of *CFTR* as a true disease gene within this region. Fine mapping of all genetic variation at this locus, and extensive functional studies, are needed to test this hypothesis because other pathomechanisms and risk genes cannot be excluded entirely. Moreover, detailed genotype–phenotype studies of Barrett's oesophagus and oesophageal adenocarcinoma, and of isolated patients with gastro-oesophageal reflux stratified for the *CFTR* risk variant, are needed that take the implicated mechanisms of gastro-oesophageal reflux in cystic fibrosis into account. This work might yield new insights in the area of Barrett's oesophagus and oesophageal adenocarcinoma research.

To our knowledge, the first risk locus to be identified that is specific to oesophageal adenocarcinoma is rs9823696 on chromosome 3q27. This SNP lies 4·9 kb downstream of the *HTR3C* gene. Highly correlated variants of this marker (*r*^2^>0·80) have been identified as regulatory active expression quantitative trait loci that affect expression of the *ABCC5* gene at this locus.^32^ However, these regulatory effects were studied in blood cells^32^ and, thus, further work needs to be done to find out if these expression quantitative trait loci are also present in tissues relevant to oesophageal adenocarcinoma. However, on the functional level, *ABCC5* represents an interesting oesophageal adenocarcinoma candidate gene. The corresponding gene product belongs to the group of ATP-binding cassette membrane proteins that play a part in energy-dependent transport of various endogenous and exogenous substrates and has been implicated in cancer development and progression.^33^, ^34^ Furthermore, as with other oesophageal adenocarcinoma genes implicated by genome-wide association studies (eg, *FOXF1* and *FOXP1*),^7^, ^9^ *ABCC5* has a role during embryonal development of the intestine.^35^ Apart from the exact functional role of rs9823696, markers that contribute solely to oesophageal adenocarcinoma development could serve as predictors for disease progression in Barrett's oesophagus. Because Barrett's oesophagus is common in the population and only a few patients develop oesophageal adenocarcinoma, specific markers for the transition of Barrett's oesophagus to oesophageal adenocarcinoma are needed. The risk locus near *HTR3C* and *ABCC5* alone accounts for only a fraction of the phenotypic variance; the OR is 1·17 between patients with oesophageal adenocarcinoma and controls, and 1·02 between individuals with Barrett's oesophagus and controls. However, identification of further oesophageal adenocarcinoma-specific markers with larger samples of patients with Barrett's oesophagus and oesophageal adenocarcinoma, together with incorporation of relevant environmental and clinical data (eg, length of Barrett's oesophagus segments, presence of low-grade dysplasia), and application of modern polygenic score approaches will help to identify patients with Barrett's oesophagus at higher risk for oesophageal adenocarcinoma. Development of such risk-prediction methods would be an important advance in clinical management, because this information could be used for more effective and individualised surveillance and intervention strategies. Since genetic data can be used for risk prediction at very early stages (eg, before development of Barrett's oesophagus), risk profiling approaches should also focus on markers that contribute solely to development of Barrett's oesophagus and are independent of the cause of gastro-oesophageal reflux.

Pathways analyses showed that cellular processes related to muscle-cell differentiation and mesenchyme development and cell differentiation are associated with development of Barrett's oesophagus and oesophageal adenocarcinoma. Involvement of the muscle-cell differentiation pathway is especially interesting because this pathway might represent a link to cellular mechanisms in the development of hiatal hernias, which have been associated with gastro-oesophageal reflux and Barrett's oesophagus.^36^, ^37^ In particular, in the most common type 1 hernia, the muscles of the oesophageal hiatus are absent or reduced to a few atrophic strands.^38^ Thus, muscle-cell differentiation pathways could have a role in formation of hiatal hernia, which in turn might increase the risk for gastro-oesophageal reflux and Barrett's oesophagus and oesophageal adenocarcinoma. By contrast, both mesenchyme-related pathways imply that the epithelial-mesenchymal transition plays a part in development of Barrett's oesophagus and oesophageal adenocarcinoma, which is characterised by loss of cell adhesion and increased cell migration and invasion. The epithelial-mesenchymal transition represents an essential step in invasion and metastasis of human cancers, particularly in early oesophageal adenocarcinoma originating from Barrett's oesophagus.^39^ However, methods used in pathways analyses can differ between studies, and results are not necessarily consistent. Thus, although the top pathways in this study are supported by the current pathophysiological ideas about Barrett's oesophagus and oesophageal adenocarcinoma, further pathways analyses and functional studies could confirm the involvement of these pathways in development of Barrett's oesophagus and oesophageal adenocarcinoma.

The only locus that showed significant heterogeneity between studies was related to SNPs within or near *TPPP* and *CEP72*. Here, the magnitude of association was larger in the Bonn study than in the other studies included in our meta-analysis. This finding points to a so-called winner's curse effect (ie, the phenomenon in which the effect size of a newly identified genetic association is overestimated because of the insufficient statistical power of the original study) in the Bonn study rather than to systematic differences between studies, because heterogeneity was only noted at this locus.

Our study has several limitations. Although we have provided bioinformatics evidence for the functional relevance of our findings, we do not provide in-vitro or in-vivo evidence for the biological function of these findings. Further studies are needed to investigate how the identified risk loci contribute to development of Barrett's oesophagus and oesophageal adenocarcinoma at the molecular and cellular level. Moreover, our study included control individuals who were not screened for the presence of Barrett's oesophagus. Although most controls were probably not affected by Barrett's oesophagus, inclusion of individuals screened for the absence of Barrett's oesophagus would have increased our power to detect further risk loci for Barrett's oesophagus and oesophageal adenocarcinoma. Furthermore, we did not include genome-wide data from a sufficiently high number of patients with isolated gastro-oesophageal reflux. Such data would have enabled us to identify risk variants that are predictive for the transition from gastro-oesophageal reflux to Barrett's oesophagus. Finally, the sample size of our study has only power for identification of risk loci with moderate effects. Although we have used the largest available sample of genome-wide association study data analysed so far from individuals with Barrett's oesophagus and oesophageal adenocarcinoma, further data from additional patients would have led to identification of more risk loci.

In conclusion, our meta-analysis identified nine new risk loci for Barrett's oesophagus and oesophageal adenocarcinoma and highlighted genes and cellular pathways likely to be implicated in disease development. To our knowledge, we have identified for the first time an oesophageal adenocarcinoma association near the *HTR3C* and *ABCC5* genes that is not observed in Barrett's oesophagus. Although the strength of genome-wide association study meta-analyses is identification of disease loci, fine-mapping and functional studies of new risk loci are now needed to reveal the disease pathophysiology. This next step—together with identification of additional risk loci using larger sample sizes through international collaborative efforts—should lead to identification of key molecules that have an important role in development of Barrett's oesophagus and oesophageal adenocarcinoma, which should finally pave the way for new molecular targets for development of advanced prevention and intervention strategies.

## Figures and Tables

**Table:** Top SNPs from loci meeting the threshold for genome-wide significance in the combined Barrett's oesophagus and oesophageal adenocarcinoma meta-analysis

	**Chromosome**	Position*	**Tested allele**	**Other allele**	**Nearest gene or region**	**INFO score**†	**Odds ratio (95% CI)**	**p**	**p for heterogeneity**
rs7255	2	20878820	T	C	*GDF7* and *LDAH*	0·92	1·14 (1·09–1·18)	9·1 × 10^−11^	0·78
rs2464469	15	58362025	A	G	*ALDH1A2*	0·97	0·89 (0·85–0·92)	4·6 × 10^−10^	0·19
rs17451754‡	7	117256712	A	G	*CFTR*	0·97	0·84 (0·80–0·89)	4·8 × 10^−10^	0·61
rs17749155‡	8	10068073	A	G	*MSRA*	0·91	1·18 (1·12–1·24)	5·2 × 10^−10^	0·77
rs10108511‡	8	11435516	T	C	*LINC00208* and *BLK*	0·98	1·12 (1·08–1·16)	2·1 × 10^−9^	0·84
rs2687202	3	70929983	T	C	*FOXP1*	0·99	1·13 (1·08–1·17)	2·3 × 10^−9^	0·92
rs1247942	12	114673723	C	G	*LOC105369996* and *TBX5*	0·98	0·89 (0·86–0·92)	2·3 × 10^−9^	0·91
rs62423175‡	6	62195368	A	G	*KHDRBS2* and *MTRNR2L9*	0·87	1·17 (1·11–1·23)	3·0 × 10^−9^	0·29
rs9918259‡	5	663092	T	C	*TPPP* and *CEP72*	0·56	1·20 (1·13–1·27)	3·2 × 10^−9^	0·037
rs9257809	6	29356331	A	G	MHC region	0·91	1·23 (1·14–1·31)	5·9 × 10^−9^	0·35
rs7852462‡	9	100310501	T	C	*TMOD1*	0·94	0·89 (0·86–0·93)	1·5 × 10^−8^	0·54
rs139606545‡	2	200045039	T	C	*SATB2*	0·98	0·90 (0·86–0·93)	2·0 × 10^−8^	0·27
rs1979654	16	86396835	C	G	*LOC732275* and *FOXF1*	0·97	0·90 (0·86–0·93)	3·3 × 10^−8^	0·29
rs199620551	19	18804294	T	TG	*CRTC1*	0·96	0·90 (0·87–0·93)	4·7 × 10^−8^	0·68

SNP=single nucleotide polymorphism.

* Position in Genome Reference Consortium human genome (build 37).

† Average of imputation quality score (INFO score) between cohorts.

‡ New risk variants at genome-wide significance level (p<5 × 10^−8^).
