# Supplementary material for: Genome-wide association studies in oesophageal adenocarcinoma and Barrett's oesophagus: a large-scale meta-analysis
Source: Lancet Oncol. 2016 Oct;17(10):1363–73. doi: 10.1016/S1470-2045(16)30240-6 (PMC5052458; doi:10.1016/S1470-2045(16)30240-6)
Supplement: Supplementary appendix [file mmc1.pdf]

THE LANCET Oncology

Supplementary appendix

This appendix formed part of the original submission and has been peer reviewed. We post it as supplied by the authors.

Supplement to: Gharahkhani P, Fitzgerald RC, Vaughan TL, et al, and the Barrett's and Esophageal Adenocarcinoma Consortium (BEACON), the Esophageal Adenocarcinoma GenEtics Consortium (EAGLE), the Wellcome Trust Case Control Consortium 2 (WTCCC2). Genome-wide association studies in oesophageal adenocarcinoma and Barrett's oesophagus: a large-scale meta-analysis. *Lancet Oncol* 2016; published online Aug 12. http://dx.doi.org/10.1016/S1470-2045(16)30240-6.

APPENDIX

First large scale meta-analysis of genome-wide association studies (GWAS) in esophageal adenocarcinoma (EA) and Barrett's esophagus (BE)

Puya Gharahkhani PhD[1*], Rebecca C. Fitzgerald MD[2*], Thomas L Vaughan MD[3*], Claire Palles PhD[4*], Prof Ines Gockel MD[5*], Ian Tomlinson MD[4], Matthew F. Buas PhD[3], Andrea May MD[6], Christian Gerges MD[7], Prof Mario Anders MD[8,9], Jessica Becker PhD[10,11], Nicole Kreuser BSc[5], Tania Noder BN[8], Marino Venerito MD[12], Lothar Veits MD[13], Thomas Schmidt MD[14], Hendrik Manner MD[15], Claudia Schmidt MD[16], Timo Hess MSc[10,11], Anne C. Böhmer PhD[10,11], Prof Jakob R. Izbicki MD[17], Prof Arnulf H. Hölscher MD[16], Prof Hauke Lang MD[18], Dietmar Lorenz MD[19], Brigitte Schumacher MD[7,20], Andreas Hackelsberger MD[21], Rupert Mayershofer MD[22], Oliver Pech MD[23], Yogesh Vashist MD[17], Katja Ott MD[14,24], Michael Vieth MD[13], Josef Weismüller MD[25], Prof Markus M. Nöthen MD[10,11], Barrett's and Esophageal Adenocarcinoma Consortium (BEACON), Esophageal Adenocarcinoma GenEtics Consortium (EAGLE), Wellcome Trust Case Control Consortium 2 (WTCCC2), Stephen Attwood MD[26], Hugh Barr MD[27], Laura Chegwidden MSc[28], John de Caestecker MD[29], Rebecca Harrison MD[30], Sharon B Love MSc[31], David MacDonald DDS[32], Paul Moayyedi MD, PhD[33], Hans Prenen MD[34], RG Peter Watson MD[35], Prasad G Iyer MD[36]; Lesley A Anderson PhD[37]; Prof Leslie Bernstein PhD[38]; Wong-Ho Chow PhD[39]; Laura J Hardie PhD[40]; Prof Jesper Lagergren PhD[41,42]; Geoffrey Liu MD[43]; Harvey A Risch MD[44]; Anna H Wu PhD[45]; Prof Weimin Ye PhD[46]; Nigel C Bird MD[47]; Nicholas J Shaheen MD[48]; Marilie D Gammon PhD[49]; Douglas A Corley MD[50,51]; Carlos Caldas PhD[52,53]; Prof Susanne Moebus PhD[54], Michael Knapp PhD[55], Wilbert H. M. Peters MD[56], Horst Neuhaus MD[7], Prof Thomas Rösch MD[8], Christian Ell MD[6], Stuart MacGregor PhD[1†], Paul Pharoah PhD[57†], Prof David C Whiteman MD[58†], Prof Janusz Jankowski MD[59,60†], Johannes Schumacher MD[10,11†]

[1]Statistical Genetics, QIMR Berghofer Medical Research Institute, Brisbane, QLD, Australia; [2]Medical Research Council (MRC) Cancer Unit, Hutchison-MRC Research Centre and University of Cambridge, Cambridge, United Kingdom; [3]Division of Public Health Sciences, Fred Hutchinson Cancer Research Center, Seattle, WA, USA; [4]Wellcome Trust Centre for Human Genetics, University of Oxford, Oxford, UK; [5]Department of Visceral, Transplant, Thoracic and Vascular Surgery, University Hospital of Leipzig, Leipzig, Germany; [6]Department of Medicine II, Sana Klinikum, Offenbach, Germany; [7]Department of Internal Medicine II, Evangelisches Krankenhaus, Düsseldorf, Germany; [8]Department of Interdisciplinary Endoscopy, University Hospital Hamburg-Eppendorf, Hamburg, Germany; [9]Department of Gastroenterology and Interdisciplinary Endoscopy, Vivantes Wenckebach-

Klinikum, Berlin, Germany; [10]Institute of Human Genetics, University of Bonn, Bonn, Germany; [11]Department of Genomics, Life & Brain Center, University of Bonn, Bonn, Germany; [12]Department of Gastroenterology, Hepatology and Infectious Diseases, Otto-von-Guericke University Hospital, Magdeburg, Germany; [13]Institute of Pathology, Klinikum Bayreuth, Bayreuth, Germany; [14]Department of General, Visceral and Transplantation Surgery, University of Heidelberg, Heidelberg, Germany; [15]Department of Internal Medicine II, HSK Hospital, Wiesbaden, Germany; [16]Department of General, Visceral and Cancer Surgery, University of Cologne, Cologne, Germany; [17]Department of General, Visceral and Thoracic Surgery, University Medical Center Hamburg-Eppendorf, University of Hamburg, Hamburg, Germany; [18]Department of General, Visceral and Transplant Surgery, University Medical Center, University of Mainz, Mainz, Germany; [19]Department of General and Visceral Surgery, Sana Klinikum, Offenbach, Germany; [20]Department of Internal Medicine and Gastroenterology, Elisabeth Hospital, Essen, Germany; [21]Gastropraxis, Wiesbaden, Germany; [22]Gastroenterologie am Burgweiher, Bonn, Germany; [23]Department of Gastroenterology and Interventional Endoscopy, St. John of God Hospital, Regensburg, Germany; [24]Department of General, Visceral and Thorax Surgery, RoMed Klinikum Rosenheim, Rosenheim, Germany; [25]Gastroenterologische Gemeinschaftspraxis, Koblenz, Germany; [26]Centre For Integrated Health Care Research, Durham University, Durham, UK; [27]Gloucestershire Royal Hospital, Gloucester, GL66NG, United Kingdom; [28]Plymouth University Peninsula School of Medicine and Dentistry, Plymouth, Devon, UK; [29]Digestive Diseases Centre, University Hospitals of Leicester, Leicester, UK; [30]Department of Cellular Pathology, Leicester Royal Infirmary, Leicester, UK; [31]Centre for Statistics in Medicine, NDORMS, University of Oxford, Oxford, UK; [32]Department of Oral Biological and Medical Sciences, University of British Columbia, Vancouver, British Columbia, Canada; [33]Department of Medicine, McMaster HC, Hamilton Ontario, Canada; [34]Department of gastroenterology, University Hospitals Gasthuisberg, Leuven, Belgium; [35]Queen's University Belfast, Centre of Medical Education, Mulhouse, Royal Victoria Hospital, Grosvenor Road BT12 6BW, N Ireland UK; [36]Division of Gastroenterology and Hepatology, Department of Internal Medicine, Mayo Clinic, Rochester, Minnesota; [37]Centre for Public Health, Queen's University Belfast, Northern Ireland, UK; [38]Department of Population Sciences, Beckman Research Institute and City of Hope Comprehensive Cancer Center, Duarte, California, USA; [39]Department of Epidemiology, MD Anderson Cancer Center, Houston, Texas, USA; [40]Division of Epidemiology, University of Leeds, Leeds, UK; [41]Department of Molecular Medicine and Surgery, Karolinska Institutet, Stockholm, Sweden; [42]Division of Cancer Studies, King's College London, United Kingdom; [43]Pharmacogenomic Epidemiology, Ontario Cancer Institute, Toronto, Ontario, Canada; [44]Department of Chronic Disease Epidemiology, Yale School of Public Health, New Haven, Connecticut, USA; [45]Department of Preventive Medicine, University of Southern California/Norris Comprehensive Cancer Center, Los Angeles, California, USA; [46]Department of Medical Epidemiology and Biostatistics, Karolinska Institute, Stockholm, Sweden; [47]Department of Oncology, Medical School, University of Sheffield, Sheffield, UK; [48]Division of Gastroenterology and Hepatology, University of North Carolina School of Medicine, University of North Carolina,

Chapel Hill, North Carolina, USA; [49]Department of Epidemiology, University of North Carolina, Chapel Hill, North Carolina, USA; [50]Division of Research, Kaiser Permanente Northern California, Oakland, California, USA; [51]San Francisco Medical Center, Kaiser Permanente Northern California, San Francisco, California, USA; [52]Department of Oncology, University of Cambridge, Cambridge, UK; [53]Cancer Research UK Cambridge Institute, University of Cambridge, Cambridge, UK; [54]Centre of Urban Epidemiology, Institute of Medical Informatics, Biometry and Epidemiology, University of Essen, Essen, Germany; [55]Institute for Medical Biometry, Informatics, and Epidemiology, University of Bonn, Bonn, Germany; [56]Department of Gastroenterology, Radboud University Nijmegen Medical Center, Nijmegen, The Netherlands; [57]Centre for Cancer Genetic Epidemiology, Department of Oncology, University of Cambridge, Cambridge, United Kingdom; [58]Cancer Control, QIMR Berghofer Medical Research Institute, Brisbane, QLD 4029, Australia; [59]University of Central Lancashire Westlakes Campus, Samuel Lindow Building, Westlakes Science and Technology Park, Moor Row, Cumbria, England; [60]Warwick Medical School, University of Warwick, Warwickshire, England. * These authors contributed equally to this work. † These authors jointly directed this work

APPENDIX: TABLE OF CONTENTS

Appendix: Material & Methods **5**
Sample description and genome-wide genotyping 5
BEACON (North America, Europe, Australia) 5
Bonn (Germany) 5
Cambridge (UK) 5
Oxford (UK) 5
Post-imputation Quality control (QC) 6
Functional analysis 6
Appendix: Results **7**
Functional annotation enrichment analysis 7
Gene-based analysis 7
Tissue enrichment analysis 7
Appendix: Figures 8
Figure 1. Meta-analysis Q-Q plots 8
Figure 2. Meta-analysis Manhattan plots 9
Figure 3. Loci with suggestive independent BE/EA association 10
Appendix: Tables **11**
Table 1. Study overview 11
Table 2. Strongest associations in the BE meta-analysis 11
Table 3. Strongest associations in the EA meta-analysis 12
Table 4. Strongest associated BE/EA SNPs in BE and EA separately 13
Table 5. Pathways for BE/EA development according to DEPICT 14
Table 6. Tissue enrichment analysis according to DEPICT 15
Table 7. The nearest genes to the most significant associated BE/EA SNPs 16
Table 8. eQTL results for BE/EA SNPs 17
Appendix: Discussion **18**
rs139606545 18
rs9918259 18
rs62423175 18
rs12207195 18
rs17749155 18
rs10108511 18
rs7852462 18
Acknowledgments **19**
References **22**

APPENDIX: MATERIAL & METHODS

The Material & Methods section comprises (i) the sample description and genome-wide genotyping separated according to the four participating sites, (ii) the quality control (QC), imputation procedure and meta-analysis as well as (iii) all follow-up analyses (gene-based analysis, pathway-based and tissue enrichment analyses, functional annotation enrichment and bioinformatics functional analyses).

Sample description and genome-wide genotyping separated for all participating sites

Barrett's and Esophageal Adenocarcinoma Consortium (BEACON) (North America, Europe, Australia): The case-control cohort used for the GWAS meta-analysis has been published previously[1]. In brief, the BEACON consortium comprises 2,413 BE cases, 1,512 EA cases and 2,185 controls, all of European descent, from 15 epidemiologic studies in North America, Europe, and Australia. Histological confirmation of BE and EA was carried out for all the participating studies. These samples were genotyped on Illumina HumanOmni1-Quad array. Informed consent was obtained from all the participants and ethics approval was obtained from the ethics boards of each participating institution. To increase the statistical power for detecting risk loci, an additional 4,541 unscreened controls of European ancestry genotyped on the Illumina HumanOmni1-Quad array were obtained from dbGaP and merged with the BEACON data. The dbGaP controls were collected from the following studies after obtaining approval from the respective committees: High Density SNP Association Analysis of Melanoma (phs000187.v1.p1), Genome-Wide Association Study of Parkinson Disease (phs000196.v2.p1), and Chronic Renal Insufficiency Study Study (CRIC) (phs000524.v1.p1). The controls from the melanoma study, also known as "MD Anderson controls", are cancer-free individuals of European ancestry who were screened for melanoma at the MD Anderson Cancer Center in Houston, Texas. The controls from the Parkinson study are Parkinson-unaffected people of European ancestry recruited at the NeuroGenetics Research Consortium-affiliated movement disorder clinics in Oregon, Washington, Georgia and New York. The renal insufficiency study includes individuals of European ancestry with measures of the estimated Glomerular Filtration Rate (eGFR). Following data cleaning and merging, 2,406 BE cases, 1,508 EA cases (3,914 BE/EA combined), and 6,718 unscreened controls were available from the BEACON study for this study (see Appendix table 1) (seven BE cases, four EA cases, and eight controls were removed due to the quality control criteria (QC); please see the QC section below).

Bonn (Germany): Following data cleaning, the sample consisted of 1,037 BE and 1,609 EA cases (2,646 BE/EA combined), as well as 3,537 controls, all of Central European descent (see Appendix table 1) (95 BE/EA cases and 62 controls were removed from the Bonn sample due to the quality control criteria). In all cases the diagnosis of BE or EA was histopathologically confirmed. Controls were a population-based sample from the Heinz Nixdorf Recall (HNR) study[2]. All participants signed informed consent and the study was approved by ethics committees from the Universities of Bonn and Leipzig (Germany). Although none of the controls were diagnosed with EA, they were not screened for BE status. Genotyping in cases and controls was performed using HumanCoreExome (235 cases, 1,786 controls), PsychArray (2,411 cases) or HumanOmniExpress BeadChips (1,751 controls) (Illumina, San Diego, USA) at the Life & Brain Center in Bonn, Germany.

Cambridge (UK): Cases of BE and EA were genotyped on the Illumina HumanOmni1-Quad array. These cases were merged with controls from the WTCCC2 including the National Blood Service (UKBS) and 1958 birth (58C) studies genotyped on custom version of the Illumina Human1.2M-Duo array. Following data cleaning and merging, 873 BE cases, 995 EA cases (1,868 BE/EA combined), and 3,408 unscreened controls, all of European descent, were available for this study (see Appendix table 1) (nine BE cases, eight EA cases, and 48 controls were removed due to the quality control criteria (QC); please see the QC section below). Barrett's esophagus cases were identified at endoscopy with a confirmed histopathological diagnosis of intestinal metaplasia from the UK Barrett's Esophagus Gene Study. Esophageal adenocarcinoma cases were selected from SOCS and had an International Classification of Diseases coding of malignant neoplasm of the esophagus (C15) and a pathological diagnosis of adenocarcinoma. All recruited participants gave informed consent, and the studies have been approved by the relevant institutional ethics review board.

Oxford (UK): The case-control cohort used for the GWAS meta-analysis has been published previously[3]. In brief, histologically confirmed BE cases from the AspECT, HANDEL and ChOPIN studies were genotyped using the Illumina 660W-Quad array. Written informed consent was obtained from all subjects. The ethics of the project were reviewed by the East London and the City Research Ethics Committee (8 June 2004, 04/Q0603/1). All UK studies were performed with national ethical committee approval (MREC numbers: AspECT 04/Q0603/1; ChOPIN/IPOD 06/Q1603/07; HANDEL 09/H0505/23; and CORGI MREC/06/Q1702/99). Cases were merged with controls from two colorectal cancer (CRC) studies CoRGI and the Colon Cancer Family Registry (Dunlop MG, Dobbins SE, Farrington SM et al. Nat Genet. 2012 44: 770–

776) and a non-overlapping set of WTCCC2 controls from the UKBS and 58C studies, also used in the Cambridge dataset. Controls were genotyped on Illumina Hap1M, Illumina Hap550, or Illumina custom Human 1.2M-Duo arrays. Following principal components analysis and data cleaning 1,851 BE cases and 3,496 controls (1,670 from NBS and 58C studies as well as 1,826 from the CRC study) were available for analysis (see Appendix table 1) (1 BE case, 0 WTCCC2 controls and 59 CRC study controls were removed due to the quality control criteria).

Post-imputation Quality control (QC)

The standard protocol for the post-imputation QC excludes SNPs with imputation quality score <0.3 and MAF<0.01. We used a slightly higher threshold for the imputation quality score (0.4 rather than 0.3) for this study to ensure that the not-well imputed SNPs were excluded. For MAF, in addition to the common variants (MAF>0.01) we also included some more rare variants (SNPs with 0.001<MAF<0.01) in this analysis. This was because we have previously shown that, in certain conditions, a pathologic rare variant (MAF ~ 0.001) could be accurately imputed and detected using a standard single variant association test[4].

Functional analysis

Bioinformatics functional analysis was performed for the genome-wide significant BE, EA and BE/EA SNPs as well as tagging SNPs using the following tools and databases: HaploReg[5], RegulomeDB[6], NIH Roadmap Epigenomics Mapping Consortium[7], SNPinfo[8], ENCODE Project Consortium[9], and eQTL-browsers including Blood eQTL-Browser[10], NCBI eQTL-Browser and GTEx-Browser[11]. Tagging SNPs were defined as markers with r^2>0.8 to the associated markers in the European 1000 Genomes Project population based on the tools HaploView[12], SNAP[13], or T1DBase[14].

APPENDIX: RESULTS

The Results section comprises all findings from the functional annotation enrichment analysis, gene-bases analysis, and tissue enrichment analysis, which are not presented in the manuscript.

Functional annotation enrichment analysis: We used the fgwas software[15] to identify annotation enrichment among the genome-wide significant loci in the BE/EA analysis. The most significant enrichment was for a region level association of gene density (top and bottom third of gene density). Ranked by significance the next most important annotations were SNP-level annotations for non-coding genes, DNase-I hypersensitivity in aortic smooth muscle and DNase-I hypersensitivity in fetal stomach. Following a model selection procedure to remove correlated annotations the model including gene density and non-coding genes was used to compute a prior distribution for the remainder of the genome outside the genome-wide significant loci. Following reweighting of the sub-threshold loci we found evidence for an additional locus at the gene *LPA* (posterior probability 0·925 compared with 0·863 without weighting by annotation).

Gene-based analysis: The genomic inflation factors in gene-based test were 1.31, 1.21 and 1.45 for BE, EA, and BE/EA analyses, respectively. We identified three additional significantly associated BE/EA loci ($P<2.8\times10^{-6}$, Bonferroni-corrected genome-wide significance threshold for 17,787 gene-based comparisons) using this approach. The significant genes are *MIR4516* on chromosome 16p13 (gene-based $P=1\times10^{-6}$), *LPAL2* on chromosome 6q25 (gene-based $P=1\times10^{-6}$), and *MFHAS1* on chromosome 8p23 (gene-based $P=2\times10^{-6}$). However, none of these loci reached gene-based genome-wide significance ($P<2.8\times10^{-6}$) after correction for genomic inflation.

Tissue enrichment analysis: We examined enrichment of gene expression in 209 tissue/cell type annotations for the genes within the BE/EA associated loci satisfying $P<5\times10^{-8}$, $P<10^{-6}$, and $P<10^{-4}$. Although we did not observe significant gene expression in gastrointestinal tract for the genes within the genome-wide significant associated regions, analyses using SNPs with $P<10^{-6}$ and $P<10^{-4}$ showed that genes within the BE/EA associated regions are highly expressed in the digestive system including the esophagus, stomach, small and large intestine, cecum and rectum (Appendix table 6). However, tissue enrichment was not significant at the Bonferroni-corrected threshold ($P<8\times10^{-5}$, considering multiple testing using the three *P*-value thresholds mentioned above and assuming that gene expression in all 209 tissue/cells is independent). The most significant result was observed for expression in the stomach (FDR<0.05, $P= 1.15\times10^{-3}$).

APPENDIX: FIGURES

Appendix Figure 1. Meta-analysis Q-Q plots.

This Figure shows the Q-Q plots for the two-sided *P*-values obtained from the GWAS meta-analysis of BE, EA, and BE/EA. The X axis shows the expected distribution of -log10(*P*-values) under the null hypothesis of no association. The Y axis shows the distribution of the observed -log10(*P*-values) in the meta-analysis. The red indicator lines show where X=Y. The genomic inflation factor lambda was 1.043, 1.005 and 1.049 for BE, EA, and BE/EA meta-analysis, respectively.

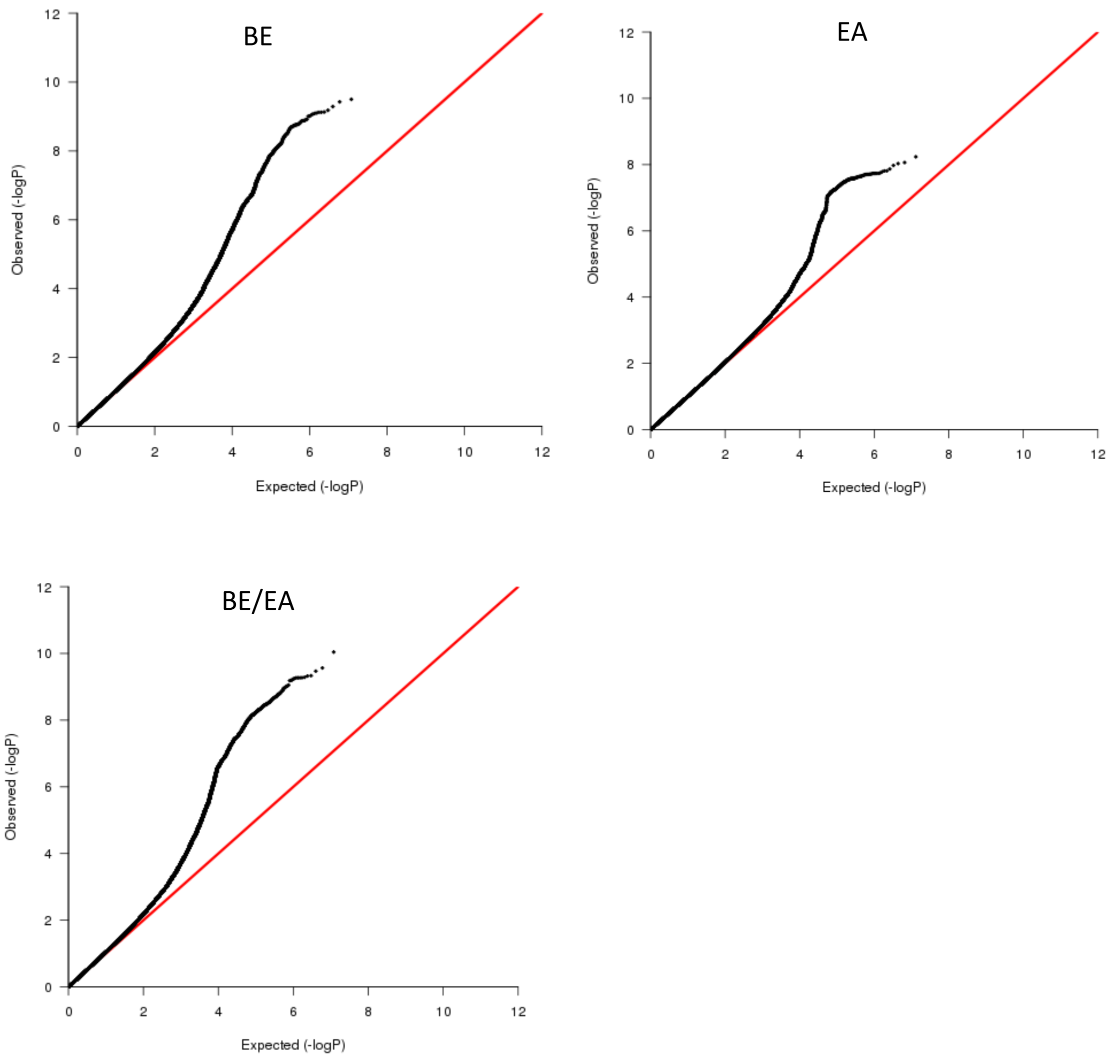

Appendix Figure 2. Meta-analysis Manhattan plots.

This Figure shows the Manhattan plots for the two-sided *P*-values obtained from GWAS meta-analysis of BE, EA and BE/EA. The SNPs have been plotted against their chromosomal positions (X axis) and the observed -log10(*P*-values) in the meta-analysis (Y axis). All SNPs on each chromosome are shown in the same colour but a distinct colour from that of the adjacent chromosome. The horizontal line in the Figure indicates the genome-wide significance level (-log10P-value=7.30).

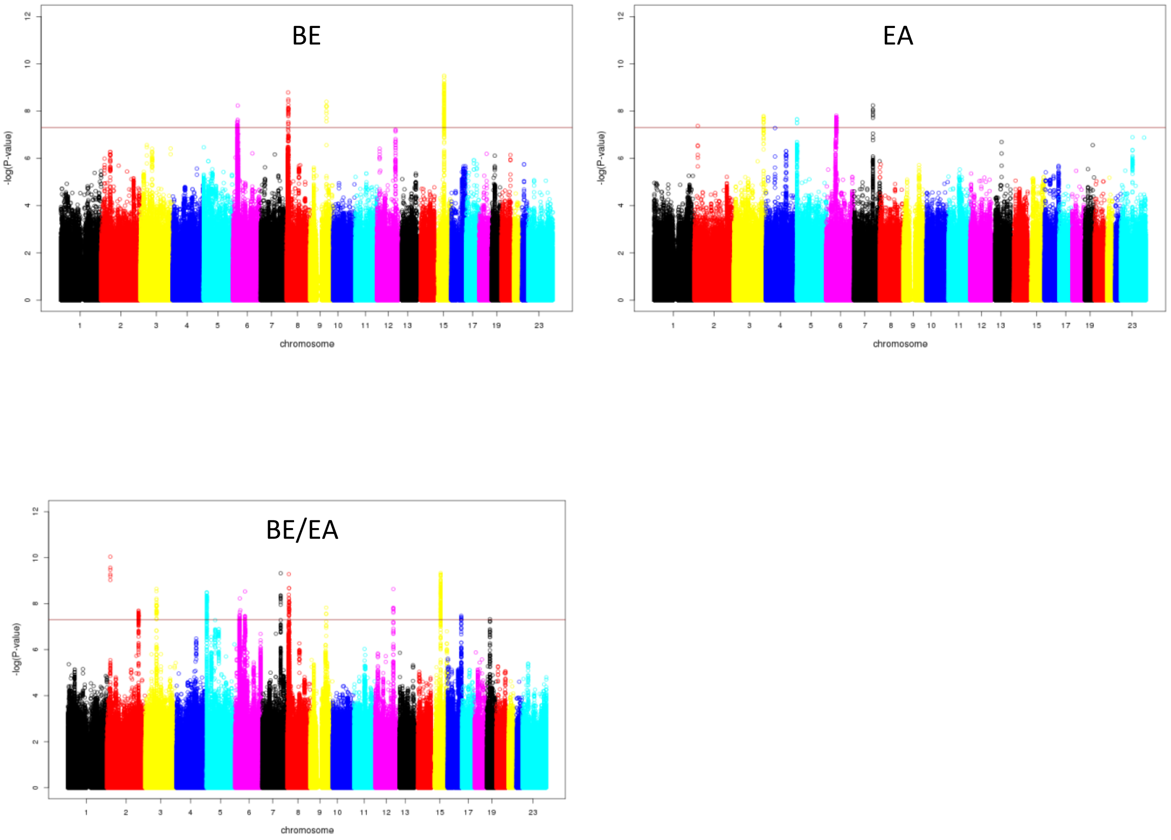

Appendix Figure 3. Regional plots for loci with suggestive independent associations in the BE/EA meta-analysis.

Plots were created after conditioning the association analysis on the most significant associated risk SNP at each locus. a) On chromosome 16q24, rs34817486 near *FOXF1-AS1*/*FOXF1* was BE/EA-associated (P=5.8×10^{-6}) conditioned on rs1979654 near *LOC732275*. b) On chromosome 5q15, rs62331139 near *LPCAT1*/*SLC6A3* was BE/EA-associated (P=7.9×10^{-6}) conditioned on rs9918259 within *TPPP*/*CEP72*.

a) b)

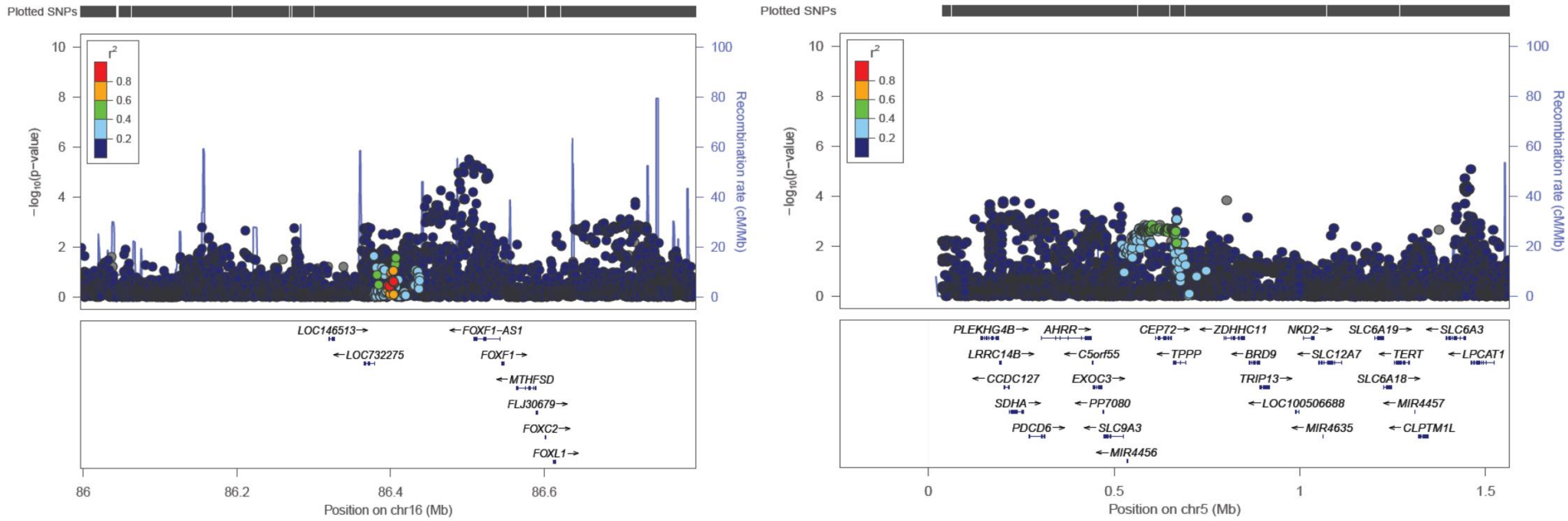

APPENDIX: TABLES

Appendix Table 1. Study overview.

Study	# of Individuals	# of males	# of females	# of BE cases	# of BE-males (%)	# of EA cases	# of EA-males (%)	# of controls	# of control-males (%)	Genotyping arrays
BEACON	10,632	7,174	3,458	2,406	1,829 (76%)	1,508	1,331 (88%)	6,718	4,014 (60%)	Illumina HumanOmni1-Quad
Bonn	6,183	3,876	2,307	1,037	697 (67%)	1,609	1,401 (87%)	3,537	1,778 (50%)	HumanCoreExome, PsychArray, HumanOmniExpress
Cambridge	5,276	3,197	2,079	873	646 (74%)	995	854 (86%)	3,408	1,697 (50%)	Illumina HumanOmni1-Quad, Illumina custom Human 1.2M-Duo array
Oxford	5,347	3,187	2,16	1,851	1,487 (80%)	-	-	3,496	1,700 (49%)	Illumina 660W-Quad, Illumina custom Human 1.2M-Duo array, Illumina Hap1M, Illumina Hap550

Appendix Table 2. Genome-wide significant associated hits in the BE meta-analysis.

SNP	Chr.	Position[a]	Effect allele	Other allele	Nearest gene	Average INFO score[b]	OR	SE(BETA)	*P*	HetPVal[c]
rs66725070	15	58,267,416	G	GACAT	*ALDH1A2*	0.981726	0.8684	0.0224	3.172e-10	0.2072
**rs17749155**	**8**	**10,068,073**	**A**	**G**	***MSRA***	**0.91099**	**1.2039**	**0.0308**	**1.642e-09**	**0.906**
**rs2409797**	**8**	**11,433,780**	**T**	**C**	***LINC00208/BLK***	**0.983657**	**1.1394**	**0.022**	**3.235e-09**	**0.9652**
**rs7852462**	**9**	**100,310,501**	**T**	**C**	***TMOD1***	**0.941289**	**0.8721**	**0.0233**	**4.03e-09**	**0.5768**
rs9257809	6	29,356,331	A	G	*MHC region*	0.75	1.2634	0.0402	5.899e-09	0.07911

[a]Position in build 37. [b]Average of imputation quality score (INFO score) between studies. [c]Heterogeneity *P*-value. Bold indicates new risk loci at genome-wide significance level ($P<5\times10^{-8}$).

Appendix Table 3. Genome-wide significant associated hits in the EA meta-analysis.

SNP	Chr.	Position[a]	Effect allele	Other allele	Nearest gene	Average INFO score[b]	OR	SE(BETA)	*P*	HetPVal[c]
**rs2188554**	**7**	**117,040,117**	**A**	**G**	***ASZ1***	**0.981801**	**1.2322**	**0.0359**	**5.809e-09**	**0.9383**
**rs76014404**	**6**	**62,391,538**	**T**	**TAAACA**	***KHDRBS2***	**0.970732**	**1.2102**	**0.0337**	**1.546e-08**	**0.3737**
**rs9823696**	**3**	**183,783,353**	**A**	**G**	***HTR3C***	**0.986525**	**1.1715**	**0.028**	**1.639e-08**	**0.6831**
**rs75783973**	**5**	**668,309**	**A**	**G**	***TPPP/CEP72***	**0.672027**	**1.3303**	**0.0511**	**2.266e-08**	**0.07026**
rs7255	2	20,878,820	T	C	*GDF7/C2orf43*	0.948708	1.1654	0.0279	4.265e-08	0.7673

[a]Position in build 37. [b]Average of imputation quality score (INFO score) between studies. [c]Heterogeneity *P*-value. Bold indicates new risk loci at genome-wide significance level ($P<5\times10^{-8}$).

Appendix Table 4. **Top SNPs from the BE/EA combined association analysis in BE and EA separately.**

					BE			EA		
**SNP**	**CHR**	**Position[a]**	**Effect allele**	**Other allele**	**OR**	**SE(BETA)**	***P***	**OR**	**SE(BETA)**	***P***
rs7255	2	20,878,820	T	C	1.1185	0.0229	1.041e-06	1.1654	0.0279	4.265e-08
rs139606545	2	200,045,039	T	C	0.9095	0.0226	2.64e-05	0.8845	0.0279	1.127e-05
rs2687202	3	70,929,983	T	C	1.1272	0.0239	5.226e-07	1.1339	0.0293	1.789e-05
rs9918259	5	663,092	T	C	1.1964	0.0352	3.427e-07	1.2037	0.0446	3.173e-05
rs9257809	6	29,356,331	A	G	1.2634	0.0402	5.899e-09	1.1411	0.0548	0.01596
rs62423175	6	62,195,368	A	G	1.1420	0.0308	1.623e-05	1.2321	0.0377	3.187e-08
rs17451754	7	117,256,712	A	G	0.8666	0.0323	8.933e-06	0.8040	0.0408	8.976e-08
rs17749155	8	10,068,073	A	G	1.2039	0.0308	1.642e-09	1.1353	0.0394	0.001264
rs10108511	8	11,435,516	T	C	1.1388	0.0221	3.834e-09	1.0814	0.0272	0.003972
rs7852462	9	100,310,501	T	C	0.8721	0.0233	4.03e-09	0.9323	0.0287	0.01453
rs1247942	12	114,673,723	C	G	0.8845	0.0228	7.356e-08	0.8971	0.0279	9.83e-05
rs2464469	15	58,362,025	A	G	0.8777	0.0226	8.248e-09	0.8987	0.0278	0.0001251
rs1979654	16	86,396,835	C	G	0.8984	0.0226	2.165e-06	0.9048	0.0278	0.0003238
rs199620551	19	18,804,294	T	TG	0.9011	0.0223	3.159e-06	0.8990	0.0277	0.0001187

[a]Position in build 37.

Appendix Table 5. Six most implicated pathways for BE/EA development according to DEPICT pathway analysis using SNPs satisfying $P<1\times10^{-4}$ from the BE/EA GWAS meta-analysis.

Gene set ID	Gene set description	Nominal P value	False discovery rate	Top genes in the gene-set (Z score)
GO:0051148	negative regulation of muscle cell differentiation	1.20e-08	<0.01	ENSG00000255399 (3.8), ENSG00000236332 (3.5), LINC00311 (3.5), DLX4 (3.4), ZDHHC11 (3.3), CRLF1 (3.3), ENSG00000225138 (3.3), ENSG00000256731 (3.3), C5orf55 (3.1), TMEM170A (2.7)
GO:0060485	mesenchyme development	4.44e-07	<0.01	ENSG00000246979 (5.0), PRDM6 (4.0), NEXN-AS1 (3.8), BARX1 (3.8), AMBN (3.5), KIF26B (3.5), TSHZ3 (3.5), ENSG00000248463 (3.4), ENSG00000255399 (3.2), DLX4 (3.1)
ENSG00000204217	BMPR2 PPI subnetwork	7.03e-07	<0.01	PRDM6 (3.9), AXIN2 (3.5), WWTR1 (3.1), FAT4 (2.9), SLC26A11 (2.8), MTMR10 (2.8), ENSG00000254300 (2.7), ENSG00000248391 (2.7), SAMD4A (2.5), CRIM1 (2.5)
GO:0048762	mesenchymal cell differentiation	9.73e-07	<0.01	ENSG00000246979 (4.2), PRDM6 (3.9), AMBN (3.5), NEXN-AS1 (3.5), AXIN2 (3.4), BARX1 (3.3), TSHZ3 (3.3), KIF26B (3.2), ENSG00000255399 (3.2), DLX4 (3.0)
GO:0010463	mesenchymal cell proliferation	1.81e-06	<0.01	ENSG00000255399 (4.9), PRDM6 (3.4), ENSG00000246979 (3.1), BARX1 (3.0), DAOA (2.8), FAT4 (2.8), ENSG00000254129 (2.7), ENSG00000216687 (2.7), ENSG00000231330 (2.7), CRLF1 (2.5)
GO:0014031	mesenchymal cell development	2.45e-06	<0.01	PRDM6 (3.4), KIF26B (3.3), AMBN (3.3), ENSG00000254235 (3.2), ENSG00000246979 (3.2), TSHZ3 (3.1), AXIN2 (3.1), ENSG00000255399 (3.0), ENSG00000248463 (2.9), BARX1 (2.9)

Appendix Table 6. Tissue enrichment analysis using DEPICT. This table shows the top tissues in which genes in the associated regions are highly expressed**.** These results were obtained using the approach implemented in DEPICT for the SNPs satisfying $P<1\times10^{-4}$ from the BE/EA GWAS meta-analysis.

**Tissue or cell type annotation**	**Name**	**More general description**	**Nominal enrichment P-value**	**False discovery rate**	**Genes highly expressed (Z score)**
A03.556.875.875	Stomach	Digestive system	1.15e-03	<0.05	RFX6 (4.3), BARX1 (3.7) , ISL1 (3.1), ENSG00000245382 (2.9) , ENSG00000246979 (2.8) ,TCF21 (2.4), MECOM (2.3) , TMPRSS3 (2.2) , MYOC (2.1), TESC (2.0)
A03.556.875	Upper Gastrointestinal Tract	Digestive System	3.07e-03	<0.20	RFX6 (3.2), BARX1 (2.3), ISL1 (2.2), MYOC (2.2), ENSG00000246979 (2.1), MECOM (2.0), ENSG00000245382 (2.0), FSIP2 (1.9), TCF21 (1.9), TMPRSS3 (1.8)
A06.407.071	Adrenal Glands	Endocrine System	4.85e-03	<0.20	ENSG00000223403 (4.8), C9orf84 (4.6), KCNQ1 (3.6), NXPH1 (3.5), ENSG00000256731 (3.2), ITGA1 (2.5), GIPC2 (2.5), DHCR7 (2.4), MSI2 (2.4), ENSG00000245382 (2.3)
A07.541	Heart	Cardiovascular System	6.36e-03	<0.20	GATA4 (5.8), XIRP1 (5.2), MTUS2 (5.1), PDE3A (5.1), FILIP1 (4.8), PDE1C (4.5), NNT (3.6), DOK7 (3.5), SAMD4A (3.4), CACNA1C (3.4)
A07.541.560	Heart Ventricles	Cardiovascular System	7.26e-03	<0.20	GATA4 (6.5), XIRP1 (5.8), MTUS2 (5.4), PDE3A (5.4), FILIP1 (5.0), PDE1C (4.9), NNT (4.0), SAMD4A (3.8), LARGE (3.7), CACNA1C (3.7)
A10.690.467	Muscle	Smooth Muscle	7.95e-03	<0.20	TRPC4 (3.1), TCF21 (2.3), DNAJB4 (2.3), DCBLD2 (2.3), ENSG00000225775 (2.3), FAM162B (2.2), POSTN (2.1), COL8A1 (1.9), CLCF1 (1.9), TSHZ3 (1.9)
A03.556.249	Lower Gastrointestinal Tract	Digestive System	8.26e-03	<0.20	ENSG00000257574 (3.4), CFTR (3.4), HOXB9 (2.9), HNF4G (2.8), AXIN2 (2.6), GIPC2 (2.1), ABHD11 (2.0), KCNQ1 (2.0), ENSG00000225953 (1.9), HOXB6 (1.9)
A03.556.249.249	Large Intestine	Digestive System	8.48e-03	<0.20	CFTR (3.5), ENSG00000257574 (3.5), HOXB9 (3.0), AXIN2 (2.8), HNF4G (2.7), GIPC2 (2.1), ABHD11 (2.1), ENSG00000225953 (2.0), KCNQ1 (2.0), HOXB6 (1.9)
A03.556.249.249.356	Colon	Digestive System	8.49e-03	<0.20	CFTR (3.5), ENSG00000257574 (3.5), HOXB9 (3.1), AXIN2 (2.8), HNF4G (2.7), GIPC2 (2.1), ENSG00000225953 (2.1), ABHD11 (2.1), KCNQ1 (2.0), HOXB6 (2.0)
A03.556.249.249.209	Cecum	Digestive System	8.71e-03	<0.20	ENSG00000257574 (3.8), CFTR (3.5), HOXB9 (3.4), HNF4G (3.3), AXIN2 (3.1), ABHD11 (2.9), RFX6 (2.5), HOXB8 (2.5), ENSG00000251676 (2.4), FUT6 (2.4)
A03.556.124	Intestines	Digestive System	9.49e-03	<0.20	CFTR (3.6), ENSG00000257574 (3.6), HNF4G (2.8), ENSG00000225953 (2.7), HOXB9 (2.7), CDKN2B-AS (2.4), AXIN2 (2.4), GIPC2 (2.3), RFX6 (2.0), FUT6 (1.9)

Appendix Table 7. The nearest genes to the most significant SNPs within each new risk locus identified in this study.

Trait	SNP[a]	Chr	Gene symbol	Gene name	Gene function
BE/EA	rs139606545	2	*SATB2*	SATB homeobox 2	This gene encodes a DNA binding protein that specifically binds nuclear matrix attachment regions. The encoded protein is involved in transcription regulation and chromatin remodelling.
EA	rs9823696	3	*HTR3C*	5-hydroxytryptamine (serotonin) receptor 3C, ionotropic	The product of this gene belongs to the ligand-gated ion channel receptor superfamily. This gene encodes subunit C of the type 3 receptor for 5-hydroxytryptamine (serotonin), a biogenic hormone that functions as a neurotransmitter, a hormone, and a mitogen. This receptor causes fast, depolarizing responses in neurons after activation. Genes encoding subunits C, D and E form a cluster on chromosome 3.
BE/EA	rs9918259	5	*TPPP/CEP72*	Tubulin polymerization promoting protein/Centrosomal protein 72kDa	TPPP: This gene may play a role in the polymerization of tubulin into microtubules, microtubule bundling and the stabilization of existing microtubules, thus maintaining the integrity of the microtubule network. It may play a role in mitotic spindle assembly and nuclear envelope breakdown.<br>CEP72: The product of this gene is a member of the leucine-rich-repeat (LRR) superfamily of proteins. The protein is localized to the centrosome, a non-membraneous organelle that functions as the major microtubule-organizing center in animal cells.
BE/EA	rs62423175	6	*KHDRBS2/MTRNR2L9*	KH Domain Containing, RNA Binding, Signal Transduction Associated 2/MT-RNR2-like 9 (pseudogene)	KHDRBS2: RNA-binding protein that plays a role in the regulation of alternative splicing and influences mRNA splice site selection and exon inclusion. Its phosphorylation by FYN inhibits its ability to regulate splice site selection. Induces an increased concentration-dependent incorporation of exon in CD44 pre-mRNA by direct binding to purine-rich exonic enhancer. May function as an adapter protein for Src kinases during mitosis. Binds both poly(A) and poly(U) homopolymers. Phosphorylation by PTK6 inhibits its RNA-binding ability (By similarity).<br>MTRNR2L9: Plays a role as a neuroprotective and antiapoptotic factor.
BE/EA	rs17451754	7	*CFTR*	Cystic fibrosis transmembrane conductance regulator	This gene encodes a member of the ATP-binding cassette (ABC) transporter superfamily. ABC proteins transport various molecules across extra- and intra-cellular membranes. ABC genes are divided into seven distinct subfamilies (ABC1, MDR/TAP, MRP, ALD, OABP, GCN20, White). This protein is a member of the MRP subfamily that is involved in multi-drug resistance. The encoded protein functions as a chloride channel and controls the regulation of other transport pathways.
BE/EA	rs17749155	8	*MSRA*	Methionine sulfoxide reductase A	This gene encodes a ubiquitous and highly conserved protein that carries out the enzymatic reduction of methionine sulfoxide to methionine. Human and animal studies have shown the highest levels of expression in kidney and nervous tissue. The protein functions in the repair of oxidatively damaged proteins to restore biological activity. Alternative splicing results in multiple transcript variants.
BE/EA	rs10108511	8	*LINC00208/BLK*	Long intergenic non-protein coding RNA 208/ BLK proto-oncogene	BLK: This gene encodes a nonreceptor tyrosine-kinase of the src family of proto-oncogenes that are typically involved in cell proliferation and differentiation. The protein has a role in B-cell receptor signaling and B-cell development. The protein also stimulates insulin synthesis and secretion in response to glucose and enhances the expression of several pancreatic beta-cell transcription factors.<br>LINC00208: is an RNA Gene, and is affiliated with the lncRNA class.
BE/EA	rs7852462	9	*TMOD1*	Tropomodulin 1	This gene encodes a member of the tropomodulin family. The encoded protein is an actin-capping protein that regulates tropomyosin by binding to its N-terminus, inhibiting depolymerization and elongation of the pointed end of actin filaments and thereby influencing the structure of the erythrocyte membrane skeleton. Multiple transcript variants encoding the same protein have been found for this gene.
BE/EA	rs12207195	6	*LPA*	Lipoprotein, Lp(A)	The protein encoded by this gene is a serine proteinase that inhibits the activity of tissue-type plasminogen activator I. The encoded protein constitutes a substantial portion of lipoprotein(a) and is proteolytically cleaved, resulting in fragments that attach to atherosclerotic lesions and promote thrombogenesis.

[a]The most significant SNP within each risk locus.

Appendix Table 8. eQTL results for the most significant SNPs within each new risk locus. This Table summarises the significant (P<0.05) eQTL results for the new risk loci identified in this study. These results were obtained from eQTL-browsers including Blood eQTL Browser, NCBI eQTL-Browser, and GTEx-Browser. Only eQTL results for the most relevant tissues have been shown.

			eQTL		
**Trait**	**SNP**[a]	**Nearest Gene**	**Tissue/cell lines**	**Gene**	***P***
BE/EA	rs139606545	*SATB2*	--	--	--
EA	rs9823696	*HTR3C*	--	--	--
BE/EA	rs9918259	*TPPP/CEP72*	Brain	TPPP	2×10^{-8}
			Lymphoblastoid cell lines	ENSG00000066230.6_476317_476493	6.3×10^{-7}
BE/EA	rs62423175	*KHDRBS2/MTRNR2L9*	--	--	--
BE/EA	rs17451754	*CFTR*	--	--	--
BE/EA	rs17749155	*MSRA*	Whole blood	MSRA	1.4×10^{-13}
			Esophagus (Mucosa)	MSRA	0.029
BE/EA	rs10108511*	*LINC00208/BLK*	Whole blood	C8orf13,C8orf12	1.3×10^{-43}
			Whole blood	BLK	1.1×10^{-36}
			Lymphoblastoid cell lines	FAM167A	7.0×10^{-17}
			Lymphoblastoid cell lines	BLK	4.3×10^{-15}
			Esophagus (Mucosa)	AF131215.9	9.4×10^{-13}
			Esophagus (Mucosa)	AF131215.2	2.2×10^{-11}
			Esophagus (Mucosa)	FAM167A	2.1×10^{-9}
			Esophagus (Muscularis)	AF131215.9	2.8×10^{-8}
			Esophagus (Muscularis)	FAM66A	4.4×10^{-8}
			Whole blood	FAM167A	7.8×10^{-8}
			Adipose - Subcutaneous	AF131215.9	7.8×10^{-8}
			Adipose - Visceral (Omentum)	AF131215.9	1.5×10^{-7}
			Whole blood	AF131215.9	4.8×10^{-7}
			Adipose - Subcutaneous	RP11-148O21.6	4.3×10^{-7}
			Lymphoblastoid cell lines	FAM86B2	4.3×10^{-7}
			Esophagus (Mucosa)	RP11-419I17.1	1.1×10^{-6}
BE/EA	rs7852462	*TMOD1*	--	--	--
BE/EA	rs12207195	*LPA*	--	--	--

[a]The most significant SNP within each risk locus.

APPENDIX: DISCUSSION

The functional annotation for *CFTR* and *HTR3C*/*ABCC5* are discussed in the main text of the paper. In the following paragraphs, we discuss the functional annotation of the remaining new identified BE/EA loci, ordered according to their chromosomal position. Appendix table 7 provides information about the nearest genes to the most significant SNPs within each new risk locus identified in this study.

rs139606545 is an intergenic SNP on chromosome 2q33 and is located ~90 kb 3' of the SATB homeobox 2 (*SATB2*) gene, which encodes a nuclear matrix-associated transcription factor. This variant is in high LD (r^2>0.80) with >130 other SNPs in a 200 kb region that extends to within 10 kb 3' of the *SATB2* locus[16]. Several of these SNPs reside in regions that are marked by active histone modifications in various tissues or are bound by transcriptional regulators. In addition, some of the correlated SNPs are eQTLs for *SATB2*[5,7]. The SATB2 protein binds to nuclear matrix attachment regions (MARs) and recruits nucleosome remodeling and histone modifier enzymes[17,18]. In addition, the gene is expressed in the normal esophagus.

rs9918259 on chromosome 5p15 lies within the 3' UTR of the tubulin polymerization promoting protein (*TPPP*) gene and is also situated within an intron of the overlapping but opposite-transcribed centrosomal protein 72kDa (*CEP72)* gene. This SNP is located within predicted miRNA binding sites[8] and could be implicated in post-transcriptional regulation of *TPPP* expression levels. The TPPP protein induces tubulin polymerization as well as microtubule bundling and plays an important role in cell proliferation[19,20]. Chromosomal gains of the implicated region have been identified in early-stage lung cancers[21] and experimental knockdown of *TPPP3* was found to inhibit tumor growth and metastasis[22]. The CEP72 protein regulates localization of centrosomal proteins and spindle formation, is often upregulated in colorectal cancers, and appears to exert oncogenic effects by counteracting the function of BRCA1/CHK2 during mitosis[23,24]. On the phenotypic level, variation at this locus has been linked to ulcerative colitis and obesity-related traits[25,26].

rs62423175 is an intergenic SNP on chromosome 6q11 located ~90 kb 5' to the MT-RNR2-like 9 pseudogene (*MTRNR2L9*). This variant modifies predicted sequence motifs for AIRE and TATA DNA binding proteins[5]. rs62423175 is highly correlated (r^2>0.80) with several other variants that are located in introns of the KH domain containing, RNA binding, signal transduction associated 2 gene (*KHDRBS2*). These SNPs modify multiple DNA regulatory motifs and are putative eQTLs for *KHDRBS2* in the brain[5]. KHDRBS2 is an RNA-binding protein and splicing regulator that is tyrosine phosphorylated by Src during mitosis[27,28].

rs12207195 on chromosome 6q25 is an intronic SNP within the lipoprotein, Lp(a) (*LPA*) gene. The encoded protein constitutes a substantial portion of lipoprotein(a) (Lp(a)). This variant is highly correlated (r^2=0.81) with a second intronic SNP, rs7767084, located 12 kb downstream[16]. rs7767084 alters predicted motifs for several transcription factors and acts as an eQTL for *LPA* in esophageal mucosa (P=0.017)[11] and in blood[5,11]. Some studies have linked LP(a) levels to cancer development[29,30].

rs17749155 on chromosome 8p23 lies within an intron of the methionine sulfoxide reductase A (*MSRA*) gene. This variant has been described as a strong eQTL for *MSRA* in whole blood (Appendix Table 8). The MSRA enzyme reduces methionine sulfoxide to methionine and functions in the repair of oxidatively damaged proteins[31]. Variation in *MSRA* has been linked to adult waist circumference (abdominal adiposity) and early-onset obesity[32,33]. Obesity and abdominal adiposity represent epidemiologic risk factors for BE and EA development[34].

rs10108511 on chromosome 8p23 lies within an intron of a long intergenic non-protein coding RNA (*LINC00208*) and in close proximity (14 kb) to the *BLK* proto-oncogene, a Src family tyrosine kinase also associated with tumor suppressor activity[35]. There is evidence for the involvement of receptor and non-receptor tyrosine kinases in molecular pathogenesis of BE and EA[36]. The variant was reported as a strong eQTL for *BLK* in blood and in lymphoblastoid cell lines as well as for other nearby genes (Appendix Table 8). In addition, rs10108511 is in moderately strong LD (r^2>0.80) with multiple other SNPs at *BLK* that have regulatory potential, likely to affect protein binding, have possible implications for miRNA binding, and are located in regions marked by active histone modifications, DNAse hypersensitivity, or POL2 recruitment.

rs7852462 on chromosome 9q22 lies within an intron of the tropomodulin 1 (*TMOD1*) gene. This variant modifies a predicted motif for STAT transcription factors and is highly correlated (r^2>0.80) with several additional intronic SNPs within ~5 kb distance[16]. These include rs7045553, which resides within a region of active histone marks and DNA hypersensitivity in multiple tissues and cell types[5]. TMOD1 is an actin-capping protein involved in the regulation of tropomyosin and the inhibition of actin filament

elongation/depolymerization[37]. TMOD1 was recently shown to be induced by NFκB signaling, elevated in triple-negative breast cancer, and associated with enhanced tumor growth[38].

Acknowledgments

The following acknowledgments are from the participating groups/networks in alphabetical order:

Barrett's and Esophageal Adenocarcinoma Consortium (BEACON) (North America, Europe, Australia). This work was supported by funding from the US National Cancer Institute *at the National Institutes of Health* (grant number R01CA136725 awarded to T.L.V and D.C.W). S. MacGregor is supported by Australian Research Council (ARC) and National Health and Medical Research Council (NHMRC) Fellowships. D.C.W is supported by a Research Fellowship (APP1058522) from the NHMRC. T.L.V is supported by an Established Investigator Award (K05CA124911) from the US National Cancer Institute. The Swedish Esophageal Cancer Study was funded by grants (R01 CA57947-03) from the National Cancer Institute and the Swedish Cancer Society (4559-B01-01XAA, 4758-B02-01XAB). The Kaiser Permanente Study was supported by US NIH grants (R01DK63616, R01CA59636) and from the California Tobacco Related Research Program (3RT-0122 and 10RT-0251). The Study of Digestive Health (Brisbane, Australia) was supported by grant number 5 RO1 CA 001833-02 from the National Cancer Institute. Its contents are solely the responsibility of the authors and do not necessarily represent the official views of the National Cancer Institute. The Australian Cancer Study was supported by Program Grants (nos. #199600 and #552429) from the National Health and Medical Research Council of Australia. The MD Anderson controls were drawn from dbGaP (study accession: phs000187.v1.p1). Genotyping of these controls were performed through the University of Texas MD Anderson Cancer Center (UTMDACC) and the Johns Hopkins University center for Inherited Disease Research (CIDR). This was supported in part by NIH grants (R01CA100264, P30CA016672, R01CA133996), the UTMDACC NIH SPORE in Melanoma (2P50CA093459) as well as by the Marit Peterson Fund for Melanoma Research. Research support to collect data and develop an application to support this project was provided by 3P50CA093459, 5P50CA097007, 5R01ES011740, and 5R01CA133996. CIDR is supported by NIH contract HHSN268200782096C. We acknowledge the principal investigators of this study, Christopher Amos, Qingyi Wei and Jeffrey E. Lee. The controls from the Genome-Wide Association Study of Parkinson Disease were obtained from dbGaP (Study Accession: phs000196.v2.p1). This work utilized in part data from the NINDS dbGaP database from the CIDR:NGRC PARKINSON'S DISEASE STUDY. This study was supported by NIH grants 5R01NS36960-10. Genotyping of these samples were performed in the Johns Hopkins University center for Inherited Disease Research (CIDR) supported by NIH contract HHSN268200782096C. We acknowledge the principal investigators and co-investigators of this study, Haydeh Payami, John Nutt, Cyrus Zabetian, Stewart Factor, Eric Molho, and Donald Higgins. The controls from the Chronic Renal Insufficiency Cohort (CRIC) were drawn from dbGaP (Study Accession: phs000524.v1.p1). The CRIC study was conducted by the CRIC investigators and supported by the National Institute of Diabetes and Digestive and Kidney Diseases (NIDDK). The data and samples from the CRIC reported here were supplied by the NIDDK Central Repositories. This manuscript was not prepared in collaboration with investigators of the CRIC study and does not necessarily reflect the opinions or views of the CRIC study, the NIDDK Central Repositories, or the NIDDK. We acknowledge the principal investigators and the project officer of this study, Harold I. Feldman, Raymond R. Townsend, Lawrence J. Appel, Mahboob Rahman, Akinlolu Ojo, James P. Lash, Jiang He, Alan S. Go, and John W. Kusek.

Members of BEACON consortium

Marilie D. Gammon[1], Douglas A. Corley[2,3], Nicholas J. Shaheen[4], Nigel C. Bird[5], Laura J. Hardie[6], Liam J. Murray[7], Brian J. Reid[8,9], Wong-Ho Chow[10], Harvey A. Risch[11], Weimin Ye[12], Geoffrey Liu[13], Leslie Bernstein[14], Prasad Iyer[15], Lesley Anderson[16], Jesper Lagergren[17,18], Rebecca Fitzgerald[19], Anna H. Wu[20], David C. Whiteman[21], Thomas L. Vaughan[8]

[1]Department of Epidemiology, University of North Carolina, Chapel Hill, North Carolina, USA; [2]Division of Research, Kaiser Permanente Northern California, Oakland, California, USA; [3]San Francisco Medical Center, Kaiser Permanente Northern California, San Francisco, California, USA; [4]Division of Gastroenterology and Hepatology, University of North Carolina School of Medicine, University of North Carolina, Chapel Hill, North Carolina, USA; [5]Department of Oncology, Medical School, University of Sheffield, Sheffield, UK; [6]Division of Epidemiology, University of Leeds, Leeds, UK; [7]Centre for Public Health, Queen's University, Belfast, Northern Ireland; [8]Division of Public Health Sciences, Fred Hutchinson Cancer Research Center, Seattle, WA, USA; [9]Division of Human Biology, Fred Hutchinson Cancer Research Center, Seattle, Washington, USA; [10]Department of Epidemiology, MD Anderson Cancer Center, Houston, Texas, USA; [11]Department of Chronic Disease Epidemiology, Yale School of Public Health, New Haven, Connecticut, USA; [12]Department of Medical

Epidemiology and Biostatistics, Karolinska Institute, Stockholm, Sweden; [13]Pharmacogenomic Epidemiology, Ontario Cancer Institute, Toronto, Ontario, Canada; [14]Department of Population Sciences, Beckman Research Institute and City of Hope Comprehensive Cancer Center, Duarte, California, USA; [15]Division of Gastroenterology and Hepatology, Department of Internal Medicine, Mayo Clinic, Rochester, Minnesota; [16]Centre for Public Health, Queen's University Belfast, Northern Ireland, UK; [17]Department of Molecular Medicine and Surgery, Karolinska Institutet, Stockholm, Sweden; [18]Division of Cancer Studies, King's College London, United Kingdom; [19]Medical Research Council (MRC) Cancer Unit, Hutchison-MRC Research Centre and University of Cambridge, Cambridge, United Kingdom; [20]Department of Preventive Medicine, University of Southern California/Norris Comprehensive Cancer Center, Los Angeles, California, USA; [21]Cancer Control, QIMR Berghofer Medical Research Institute, Brisbane, QLD 4029, Australia

Bonn (Germany). This work was supported by funding from the Else Kröner Fresenius Stiftung (EKFS) (grant number 2013_A118 awarded to I.G. and J.S.). M.M.N. is a member of the DFG funded Excellence Cluster ImmunoSensation. The Heinz Nixdorf Recall cohort was established with the generous support of the Heinz Nixdorf Foundation, Germany.

Cambridge (UK). The UK Barrett's oesophagus gene study was funded by a Medical Research Council Programme grant. The UK SOCS study was funded by CRUK as well as funding from the Cambridge NIHR biomedical research centre and the Cambridge Experimental Cancer Medicine Centre. Genotyping of Cambridge samples was supported by funding from the US National Cancer Institute at the National Institutes of Health (grant number R01CA136725 awarded to T.L.V and D.C.W). This study made use of data generated by the Wellcome Trust Case Control Consortium: Funding for the project was provided by the Wellcome Trust under award 076113; a full list of the investigators who contributed to the generation of the data is available from the website (http://www.wtccc.org.uk/).

Oxford (UK). This work was supported by the Esophageal Adenocarcinoma GenE Consortia incorporating the ChOPIN project (grant C548/A5675), the Inherited Predisposition of neoplasia analysis of genomic DNA (IPOD) from AspECT and BOSS clinical trials project (grant MGAG1G7R), Cancer Research UK (AspECT, grants C548/A4584 and D9612L00090), the Histological AssessmeNt Determining EpitheliaL Response (HANDEL) (grant C548/A9085), the AstraZeneca UK educational grant, the University Hospitals of Leicester R and D grant, and AspECT (T91 5211 University of Oxford grant HDRMJQ0).

Membership of Wellcome Trust Case Control Consortium 2

Management Committee

Peter Donnelly (Chair)[1,2], Ines Barroso (Deputy Chair)[3], Jenefer M Blackwell[4, 5], Elvira Bramon[6] , Matthew A Brown[7] , Juan P Casas[8] , Aiden Corvin[9], Panos Deloukas[3], Audrey Duncanson[10], Janusz Jankowski[11], Hugh S Markus[12], Christopher G Mathew[13], Colin NA Palmer[14], Robert Plomin[15], Anna Rautanen[1], Stephen J Sawcer[16], Richard C Trembath[13], Ananth C Viswanathan[17], Nicholas W Wood[18]

Data and Analysis Group

Chris C A Spencer[1], Gavin Band[1], Céline Bellenguez[1], Colin Freeman[1], Garrett Hellenthal[1], Eleni Giannoulatou[1], Matti Pirinen[1], Richard Pearson[1], Amy Strange[1], Zhan Su[1], Damjan Vukcevic[1], Peter Donnelly[1,2]

DNA, Genotyping, Data QC and Informatics Group

Cordelia Langford[3], Sarah E Hunt[3], Sarah Edkins[3], Rhian Gwilliam[3], Hannah Blackburn[3], Suzannah J Bumpstead[3], Serge Dronov[3], Matthew Gillman[3], Emma Gray[3], Naomi Hammond[3], Alagurevathi Jayakumar[3], Owen T McCann[3], Jennifer Liddle[3], Simon C Potter[3], Radhi Ravindrarajah[3], Michelle Ricketts[3], Matthew Waller[3], Paul Weston[3], Sara Widaa[3], Pamela Whittaker[3], Ines Barroso[3], Panos Deloukas[3].

Publications Committee

Christopher G Mathew (Chair)[13], Jenefer M Blackwell[4,5], Matthew A Brown[7], Aiden Corvin[9], Chris C A Spencer[1]

1) Wellcome Trust Centre for Human Genetics, University of Oxford, Roosevelt Drive, Oxford OX3 7BN, UK; 2) Dept Statistics, University of Oxford, Oxford OX1 3TG, UK; 3) Wellcome Trust Sanger Institute, Wellcome Trust Genome Campus, Hinxton, Cambridge CB10 1SA, UK; 4) Telethon Institute for Child Health Research,

Centre for Child Health Research, University of Western Australia, 100 Roberts Road, Subiaco, Western Australia 6008; 5) Cambridge Institute for Medical Research, University of Cambridge School of Clinical Medicine, Cambridge CB2 0XY, UK; 6) Department of Psychosis Studies, NIHR Biomedical Research Centre for Mental Health at the Institute of Psychiatry, King's College London and The South London and Maudsley NHS Foundation Trust, Denmark Hill, London SE5 8AF, UK; 7) University of Queensland Diamantina Institute, Brisbane, Queensland, Australia; 8) Dept Epidemiology and Population Health, London School of Hygiene and Tropical Medicine, London WC1E 7HT and Dept Epidemiology and Public Health, University College London WC1E 6BT, UK; 9) Neuropsychiatric Genetics Research Group, Institute of Molecular Medicine, Trinity College Dublin, Dublin 2, Eire; 10) Molecular and Physiological Sciences, The Wellcome Trust, London NW1 2BE; 11) Department of Oncology, Old Road Campus, University of Oxford, Oxford OX3 7DQ, UK , Digestive Diseases Centre, Leicester Royal Infirmary, Leicester LE7 7HH, UK and Centre for Digestive Diseases, Queen Mary University of London, London E1 2AD, UK; 12) Clinical Neurosciences, St George's University of London, London SW17 0RE; 13) King's College London Dept Medical and Molecular Genetics, King's Health Partners, Guy's Hospital, London SE1 9RT, UK; 14) Biomedical Research Centre, Ninewells Hospital and Medical School, Dundee DD1 9SY, UK; 15) King's College London Social, Genetic and Developmental Psychiatry Centre, Institute of Psychiatry, Denmark Hill, London SE5 8AF, UK; 16) University of Cambridge Dept Clinical Neurosciences, Addenbrooke's Hospital, Cambridge CB2 0QQ, UK; 17) NIHR Biomedical Research Centre for Ophthalmology, Moorfields Eye Hospital NHS Foundation Trust and UCL Institute of Ophthalmology, London EC1V 2PD, UK; 18) Dept Molecular Neuroscience, Institute of Neurology, Queen Square, London WC1N 3BG, UK.

Members of Esophageal Adenocarcinoma GenEtics Consortium (EAGLE)

Peter Isaacs[1], Conrad Beckett[2], Sue Cullen[3], David Hobday[4], Ameet Dhar[5], Deepak Kejariwal[6], James Shutt[7], Ian Sargeant[8], Konrad Koss[9], Charles Grimley[10], Hugh Barr[11], Helen Winter[12], Andrew Dixon[13] ,Hugh McMurty[14], Matthew Johnson[15], Haythem Ali[16], Sandro Lazon-Miller[17], Stuart Paterson[18], Ian Beales[19], Chris MacDonald[20], Matt Rutter[21], Alex Moran[22], Chris Haig[23], Krish Ragunath[24], Siba Senapati[25], Pradeep Bhandari[26], Saj Wajed[27], Salma Alam[28], Mark Farrent[29], Yeng Ang[30], Nigel Trudgill[31], Mark Smith[32], Keith George[33], Arvind Ramadas[34], Simon Panter[35], Vinod Patel[36], Laurence Lovat[37], Mark Kelly[38], Praful Patel[39], Stephen Falk[40], Chuka Nwokolo[41], John deCaestecker[42], Subramaniam Ramakrishnan[43], Yang Eng[44], Sean Kelly[45], Art Tucker[46], Paul Mullins[47], Hans Prenen[48], Janusz Jankowski[49]

[1]Blackpool Victoria Hospital, Blackpool, UK; [2]Bradford Royal Infirmary, Bradford, UK; [3]Wycombe General Hospital, High Wycombe' UK; [4]City Hospitals, Sunderland, UK; [5]Countess of Chester Hospital, Chester,UK; [6]University Hospital of North Durham, County Durham, UK; [7]Dorset County Hospital, [8]Lister Hospital & QE II Hospital, Welwyn, UK; [9]Macclesfield District General Hospital, Macclesfield, UK; [10]Burnley General Hospital & Royal Blackburn, Burnley, Lancashire, UK; [11]Gloucestershire Royal Hospital, Gloucester, UK; [12]Great Western Hospital, Swindon, UK; [13]Kettering General Hospital' Kettering, UK; [14]Royal Preston Hospital, Preston, UK; [15]Luton & Dunstable Hospital, Luton, UK; [16]Maidstone Hospital, Maidstone, UK; [17]Milton Keynes Hospital' Milton Keynes, UK; [18]Forth Valley, Larbert, UK; [19]Norfolk and Norwich University Hospital, Colney Ln, Norwich, UK; [20]Cumberland Infirmary, Carlisle, UK; [21]University Hospital of North Tees, Stockton-on-Tees, Cleveland, UK; [22]North Devon District Hospital, North Devon, UK; [23]Wansbeck General Hospital, Ashington, UK; [24]Queens Medical Centre, Nottingham, UK; [25]Royal Oldham Infirmary, North Manchester General Hospital & Rochdale Infirmary, UK; [26]Queen Alexandra Hospital, Portsmouth, UK; [27]Royal Devon and Exeter Hospital, Exeter, UK; [28]Royal Marsden Hospital, Surrey, UK; [29]Royal United Hospital Bath, Bath, UK; [30]Salford Royal Hospital, Stott Ln, Salford, UK; [31]Sandwell General Hospital Lyndon, UK; [32]Royal Shrewsbury Hospital, Shrewsbury, UK; [33]Torbay Hospital, Torquay, UK; [34]James Cook University Hospital, Middlesbrough, UK; [35]South Tyneside District Hospital, Southshields, UK; [36]Tameside General Hospital, Ashton-under-Lyne, Lancashire, UK; [37]University College London Hospitals NHS foundation trust, London, UK; [38]Wythenshawe Hospital, Manchester, UK; [39]Southampton General Hospital, Southampton, UK; [40]Bristol Haematology and Oncology Centre, Bristol,UK; [41]University Hospital Coventry, Coventry,UK; [42]Leicester Royal Infirmary, Leicester,UK; [43]Warrington Hospital, Warrington, UK; [44]Royal Albert Edward Infirmary, Wigan, [45]York Teaching Hospital, York, North Yorkshire, UK; [46]St Bartholomews Hospital London (Ethics), London, UK; [47]Prince George Hospital British Columbia (Overseas Advisor), Prince George, BC, Canada; [48]University Hospitals Gasthuisberg, Leuven, Belgium (Overseas Advisor), [49]University of Central Lancashire Medical School Preston,Lancashire, UK

References

1. Levine DM, Ek WE, Zhang R, et al. A genome-wide association study identifies new susceptibility loci for esophageal adenocarcinoma and Barrett's esophagus. *Nature genetics* 2013; **45**(12): 1487-93.
2. Schmermund A, Mohlenkamp S, Stang A, et al. Assessment of clinically silent atherosclerotic disease and established and novel risk factors for predicting myocardial infarction and cardiac death in healthy middle-aged subjects: rationale and design of the Heinz Nixdorf RECALL Study. Risk Factors, Evaluation of Coronary Calcium and Lifestyle. *American heart journal* 2002; **144**(2): 212-8.
3. Palles C, Chegwidden L, Li X, et al. Polymorphisms near TBX5 and GDF7 are associated with increased risk for Barrett's esophagus. *Gastroenterology* 2015; **148**(2): 367-78.
4. Gharahkhani P, Burdon KP, Hewitt AW, et al. Accurate Imputation-Based Screening of Gln368Ter Myocilin Variant in Primary Open-Angle Glaucoma. *Investigative ophthalmology & visual science* 2015; **56**(9): 5087-93.
5. Ward LD, Kellis M. HaploReg: a resource for exploring chromatin states, conservation, and regulatory motif alterations within sets of genetically linked variants. *Nucleic acids research* 2012; **40**(Database issue): D930-4.
6. Boyle AP, Hong EL, Hariharan M, et al. Annotation of functional variation in personal genomes using RegulomeDB. *Genome research* 2012; **22**(9): 1790-7.
7. Bernstein BE, Stamatoyannopoulos JA, Costello JF, et al. The NIH Roadmap Epigenomics Mapping Consortium. *Nature biotechnology* 2010; **28**(10): 1045-8.
8. Xu Z, Taylor JA. SNPinfo: integrating GWAS and candidate gene information into functional SNP selection for genetic association studies. *Nucleic acids research* 2009; **37**(Web Server issue): W600-5.
9. ENCODE_Project_Consortium. An integrated encyclopedia of DNA elements in the human genome. *Nature* 2012; **489**(7414): 57-74.
10. Westra HJ, Peters MJ, Esko T, et al. Systematic identification of trans eQTLs as putative drivers of known disease associations. *Nature genetics* 2013; **45**(10): 1238-43.
11. GTEx_Consortium. Human genomics. The Genotype-Tissue Expression (GTEx) pilot analysis: multitissue gene regulation in humans. *Science (New York, NY)* 2015; **348**(6235): 648-60.
12. Barrett JC, Fry B, Maller J, Daly MJ. Haploview: analysis and visualization of LD and haplotype maps. *Bioinformatics (Oxford, England)* 2005; **21**(2): 263-5.
13. Johnson AD, Handsaker RE, Pulit SL, Nizzari MM, O'Donnell CJ, de Bakker PI. SNAP: a web-based tool for identification and annotation of proxy SNPs using HapMap. *Bioinformatics (Oxford, England)* 2008; **24**(24): 2938-9.
14. Burren OS, Adlem EC, Achuthan P, Christensen M, Coulson RM, Todd JA. T1DBase: update 2011, organization and presentation of large-scale data sets for type 1 diabetes research. *Nucleic acids research* 2011; **39**(Database issue): D997-1001.
15. Pickrell JK. Joint analysis of functional genomic data and genome-wide association studies of 18 human traits. *Am J Hum Genet* 2014; **94**(4): 559-73.
16. Abecasis GR, Auton A, Brooks LD, et al. An integrated map of genetic variation from 1,092 human genomes. *Nature* 2012; **491**(7422): 56-65.
17. Britanova O, Akopov S, Lukyanov S, Gruss P, Tarabykin V. Novel transcription factor Satb2 interacts with matrix attachment region DNA elements in a tissue-specific manner and demonstrates cell-type-dependent expression in the developing mouse CNS. *The European journal of neuroscience* 2005; **21**(3): 658-68.
18. Gyorgy AB, Szemes M, de Juan Romero C, Tarabykin V, Agoston DV. SATB2 interacts with chromatin-remodeling molecules in differentiating cortical neurons. *The European journal of neuroscience* 2008; **27**(4): 865-73.
19. Vincze O, Tokesi N, Olah J, et al. Tubulin polymerization promoting proteins (TPPPs): members of a new family with distinct structures and functions. *Biochemistry* 2006; **45**(46): 13818-26.

20. Zhou W, Wang X, Li L, et al. Depletion of tubulin polymerization promoting protein family member 3 suppresses HeLa cell proliferation. *Molecular and cellular biochemistry* 2010; **333**(1-2): 91-8.
21. Kang JU, Koo SH, Kwon KC, Park JW, Kim JM. Gain at chromosomal region 5p15.33, containing TERT, is the most frequent genetic event in early stages of non-small cell lung cancer. *Cancer genetics and cytogenetics* 2008; **182**(1): 1-11.
22. Zhou W, Li J, Wang X, Hu R. Stable knockdown of TPPP3 by RNA interference in Lewis lung carcinoma cell inhibits tumor growth and metastasis. *Molecular and cellular biochemistry* 2010; **343**(1-2): 231-8.
23. Oshimori N, Li X, Ohsugi M, Yamamoto T. Cep72 regulates the localization of key centrosomal proteins and proper bipolar spindle formation. *Embo j* 2009; **28**(14): 2066-76.
24. Luddecke S, Ertych N, Stenzinger A, et al. The putative oncogene CEP72 inhibits the mitotic function of BRCA1 and induces chromosomal instability. *Oncogene* 2015: 1-9.
25. McGovern DP, Gardet A, Torkvist L, et al. Genome-wide association identifies multiple ulcerative colitis susceptibility loci. *Nature genetics* 2010; **42**(4): 332-7.
26. Comuzzie AG, Cole SA, Laston SL, et al. Novel genetic loci identified for the pathophysiology of childhood obesity in the Hispanic population. *PloS one* 2012; **7**(12): e51954.
27. Di Fruscio M, Chen T, Richard S. Characterization of Sam68-like mammalian proteins SLM-1 and SLM-2: SLM-1 is a Src substrate during mitosis. *Proceedings of the National Academy of Sciences of the United States of America* 1999; **96**(6): 2710-5.
28. Haegebarth A, Heap D, Bie W, Derry JJ, Richard S, Tyner AL. The nuclear tyrosine kinase BRK/Sik phosphorylates and inhibits the RNA-binding activities of the Sam68-like mammalian proteins SLM-1 and SLM-2. *The Journal of biological chemistry* 2004; **279**(52): 54398-404.
29. Mieno MN, Sawabe M, Tanaka N, et al. Significant association between hypolipoproteinemia(a) and lifetime risk of cancer: an autopsy study from a community-based Geriatric Hospital. *Cancer Epidemiol* 2014; **38**(5): 550-5.
30. Marrer E, Wagner A, Montaye M, et al. Lipoprotein(a) plasma levels and the risk of cancer: the PRIME study. *Eur J Cancer Prev* 2013; **22**(3): 286-93.
31. Moskovitz J, Bar-Noy S, Williams WM, Requena J, Berlett BS, Stadtman ER. Methionine sulfoxide reductase (MsrA) is a regulator of antioxidant defense and lifespan in mammals. *Proceedings of the National Academy of Sciences of the United States of America* 2001; **98**(23): 12920-5.
32. Lindgren CM, Heid IM, Randall JC, et al. Genome-wide association scan meta-analysis identifies three Loci influencing adiposity and fat distribution. *PLoS genetics* 2009; **5**(6): e1000508.
33. Scherag A, Dina C, Hinney A, et al. Two new Loci for body-weight regulation identified in a joint analysis of genome-wide association studies for early-onset extreme obesity in French and german study groups. *PLoS genetics* 2010; **6**(4): e1000916.
34. Reid BJ, Li X, Galipeau PC, Vaughan TL. Barrett's oesophagus and oesophageal adenocarcinoma: time for a new synthesis. *Nature reviews Cancer* 2010; **10**(2): 87-101.
35. Zhang H, Peng C, Hu Y, et al. The Blk pathway functions as a tumor suppressor in chronic myeloid leukemia stem cells. *Nature genetics* 2012; **44**(8): 861-71.
36. Clemons NJ, Phillips WA, Lord RV. Signaling pathways in the molecular pathogenesis of adenocarcinomas of the esophagus and gastroesophageal junction. *Cancer Biol Ther* 2013; **14**(9): 782-95.
37. Fritz-Six KL, Cox PR, Fischer RS, et al. Aberrant myofibril assembly in tropomodulin1 null mice leads to aborted heart development and embryonic lethality. *The Journal of cell biology* 2003; **163**(5): 1033-44.
38. Ito-Kureha T, Koshikawa N, Yamamoto M, et al. Tropomodulin 1 expression driven by NF-kappaB enhances breast cancer growth. *Cancer research* 2015; **75**(1): 62-72.
